# Neurofilament light chain improves clinical prognostic models for Guillain-Barré syndrome

**DOI:** 10.1136/jnnp-2025-336046

**Published:** 2025-05-02

**Authors:** Robin C M Thomma, Linda W G Luijten, Sander J van Tilburg, Eveline J A Wiegers, Charlotte E Teunissen, Lisa Vermunt, Pieter A van Doorn, Ruth Huizinga, Bart C Jacobs

**Affiliations:** 1 Department of Neurology, Erasmus MC University Medical Center Rotterdam, Rotterdam, The Netherlands; 2 Department of Immunology, Erasmus MC University Medical Center Rotterdam, Rotterdam, The Netherlands; 3 Neurochemistry Laboratory, Department of Clinical Chemistry, Amsterdam Neuroscience, Amsterdam University Medical Centres, Amsterdam, The Netherlands

**Keywords:** GUILLAIN-BARRE SYNDROME, Patient Outcome Assessment, Prognosis

## Abstract

**ABSTRACT:**

**Background:**

Several prognostic models predict clinical outcomes in Guillain-Barré syndrome (GBS). Recently, neurofilament light chain (NfL) has emerged as a prognostic biomarker. We investigated the added prognostic value of NfL in serum (sNfL) and cerebrospinal fluid (cNfL) to models based on clinical factors predicting respiratory failure and inability to walk in GBS.

**Methods:**

We included patients from a randomised placebo-controlled trial (second intravenous immunoglobulin dose in GBS). Serum was acquired at entry and week 1, 2, 4 and 12 and cerebrospinal fluid at entry. NfL levels were determined on a single molecule array. The additional prognostic value of NfL to the (modified) Erasmus GBS Outcome Score ((m)EGOS) and (modified) Erasmus GBS Respiratory Insufficiency Score was evaluated using logistic regression analyses.

**Results:**

In total, 293 patients were included (74 (25%) mechanically ventilated, 38/275 (13%) unable to walk at 26 weeks). Higher sNfL at entry, week 1 and week 2 and cNfL at entry were associated with inability to walk at 4 and 26 weeks. Neither sNfL nor cNfL levels at entry were associated with respiratory failure. The EGOS and mEGOS improved after adding NfL (∆C-statistic range: 0.01–0.11), especially the models predicting outcome at 26 weeks. A new model predicting inability to walk at 26 weeks consisting of sNfL at entry, GBS disability score at entry and Medical Research Council sum score at week 2 performed best (C-statistic: 0.88 (95% CI 0.83 to 0.94)).

**Conclusions:**

Addition of NfL may improve clinical prognostic models for the prediction of inability to walk, but not of respiratory failure.

**Trial registration number:**

NTR2224/NL2107.

**WHAT IS ALREADY KNOWN ON THIS TOPIC**

**WHAT THIS STUDY ADDS**

- We showed that NfL levels in serum and CSF both have an additional prognostic value to existing clinical prognostic models predicting the inability to walk in GBS, especially at 26 weeks, but not to models predicting respiratory insufficiency, and we developed a new prognostic model based on NfL levels and clinical predictors.

**HOW THIS STUDY MIGHT AFFECT RESEARCH, PRACTICE OR POLICY**

- The new prognostic model is important to improve outcome prediction in GBS, although validation studies are required before use in practice. Early prognostication is increasingly relevant not only to inform patients and prepare clinicians but also for selecting patients for clinical trials, conducting observational treatment studies and enabling prognostic stratification in research.

## Introduction

 Guillain-Barré syndrome (GBS) is an acute immune-mediated polyradiculoneuropathy with a highly variable clinical course and outcome.^1^ GBS is characterised by progressive limb weakness and hyporeflexia or areflexia, and varying concomitant symptoms caused by cranial, sensory and/or autonomic nerve dysfunction.^1^ Some patients have mild leg weakness only, while others develop tetraparesis and respiratory failure.^2^ Moreover, a subset of patients may recover spontaneously, but up to 20% of patients remain unable to walk 6 months after onset.^1^ Due to the clinical heterogeneity, outcome prediction has been a major challenge in the care for patients with GBS.

Several clinical prognostic models have been developed and validated for early outcome prediction.^3^,^6^ The (modified) Erasmus GBS Outcome Score (EGOS/mEGOS) and the (modified) Erasmus GBS Respiratory Insufficiency Score (EGRIS/mEGRIS) are recommended for use in clinical practice by recent guidelines.^7 8^ The EGOS predicts the inability to walk at 26 weeks after disease onset based on age, preceding diarrhoea and the GBS Disability Score (GBS-DS) at 2 weeks after hospital admission.^3^ In the mEGOS, the GBS-DS was replaced by the Medical Research Council (MRC) sum score at hospital admission or week 1.^4^ The EGRIS predicts respiratory failure in the first week based on the number of days since onset of weakness, presence of facial or bulbar weakness and the MRC sum score at hospital admission.^5^ This model was simplified into the mEGRIS, in which the latter two prognostic factors were replaced by the presence of bulbar weakness and MRC scores for neck flexion and bilateral hip flexion at hospital admission.^6^ The accuracy of these models has been validated in international patient cohorts, and they are frequently used for outcome prediction in clinical practice.^9^,^11^

Currently, prognostication in GBS is based only on clinical features. This might be improved by incorporating biomarkers in clinical practice.^12^ Neurofilament light chain (NfL), a structural neuronal protein that—when present in serum or cerebrospinal fluid (CSF)—is highly specific for neuro-axonal damage, is a promising biomarker for outcome prediction for various neurological disorders, including GBS.^12 13^ Several studies have investigated the prognostic value of serum NfL (sNfL) in GBS and showed that baseline NfL in serum or plasma is increased in GBS, and levels are associated with the disease course and outcomes.^14^,^19^ In addition, several studies have demonstrated that NfL levels in CSF (cNfL) are also increased in GBS, especially in severe forms with poor recovery, probably in part reflecting injury of nerve roots.^14 17 20 21^

At present, it is unknown whether NfL in either matrix can be used to improve the performance of clinical prognostic models. The aim of this study was to investigate the incremental prognostic value of sNfL and cNfL to clinical prognostic models predicting inability to walk and respiratory failure in GBS.

## Methods

### Study population and data collection

Patients with GBS who previously participated in a Dutch national multicentre (59 hospitals) randomised placebo-controlled trial were included. The trial investigated the efficacy of a second intravenous immunoglobulin (IVIg) dose in patients with a poor prognosis (SID-GBS trial).^22^ Patients aged 12 years or older, fulfilling the diagnostic criteria for GBS, with an indication to receive treatment with a standard course of IVIg (2 g/kg in 5 days) within the first 2 weeks from onset of weakness, were eligible for inclusion. Exclusion criteria were a known allergy to matched blood or plasma products, pregnancy, breastfeeding, a selective IgA deficiency, clinical evidence of polyneuropathy resulting from other causes, immunosuppressive treatment in the last month, severe concurrent disease and the inability to complete 6 months of follow-up. For analyses in the current study, patients with chronic inflammatory demyelinating polyneuropathy were excluded.

Following a standard dose of IVIg, the prognosis of each patient was predicted based on the mEGOS at day 7. Patients with a poor prognosis (mEGOS of 6–12) were randomised to receive a second IVIg dose (2 g/kg in 5 days) or placebo (albumin, 0.32 g/kg in 5 days) at 7–9 days after the first standard dose. Patients with a good prognosis were not randomised. Both randomised and non-randomised patients were included in the current study. Clinical data and serum were collected at study entry and after 1, 2, 4 and 12 weeks of follow-up, with additional clinical data collection at 26 weeks. CSF was acquired at study entry.

The trial was registered in the Netherlands Trial Register (NTR2224/NL2107).

### Determination of NfL levels

Serum and CSF were stored at −80°C until use. Measurement of sNfL and cNfL was performed batch-wise at a single laboratory (Neurochemistry Lab Amsterdam) using the NF-Light Advantage Kit (Quanterix; Billerica, Massachusetts, USA) on a Single Molecule Array (HD-X Analyzer; Quanterix) by researchers blinded for the disease course and outcome. Further details on procedures used to determine final NfL levels and interassay and intra-assay variabilities have been described previously.^18^

### Data processing

To perform complete case analyses, four subcohorts containing only patients with an available serum or CSF sample at a given time point were established (serum: study entry, week 1, week 2; CSF: study entry). NfL data from study entry (pretreatment), week 1 (5–12 days) and week 2 (12–21 days) were used for analyses due to their clinical relevance for early prediction of the inability to walk 10 m unaided during follow-up (4 and 26 weeks). However, only data from study entry were deemed relevant for predicting respiratory failure (patients requiring mechanical ventilation), due to its generally early onset within the first week.^6^ NfL data from week 4 and week 12 were not used for analyses, since they were not considered to be relevant for early outcome prediction for either of the investigated outcomes.

To increase statistical power, missing data from clinical variables included in investigated clinical prognostic models and missing NfL data from entry, week 1 and week 2 were imputed by single imputation using the ‘mice’ package (V.3.16.0) in R statistical software (V.2023.12.0). Applied imputation procedures are described in detail in online supplemental file 1. Since imputed data are not observed data and might therefore introduce uncertainties, analyses performed in imputed data were also performed in non-imputed data for validation.

Based on the distribution of NfL levels and comparisons between models containing log-transformations, fractional polynomials or restricted cubic splines, levels were log-transformed (natural log) for imputations and analyses (except for descriptive analyses). In addition, sNfL values were converted to Z-scores for age-standardised subanalyses.^23^ No equation for Z-score conversion of cNfL is currently available.

### Statistical analyses

Comparative analyses between groups were performed using χ^2^ and Fisher’s exact tests for nominal variables and Mann-Whitney U and Kruskal-Wallis tests for continuous variables. Correlations were investigated with the Spearman correlation coefficient.

Univariable logistic regression analyses were employed to validate associations of individual components included in each clinical prognostic model with their respective outcome and to explore associations of sNfL and cNfL with outcomes. Analyses were performed on non-imputed data from the complete SID-GBS trial cohort. Investigated variables were individual components included in the EGRIS, mEGRIS, EGOS and mEGOS.^3^,^6^ Associations were expressed as ORs (binomial regression) and risk ratios (modified Poisson regression with SEs corrected using a sandwich variance estimator) along with their 95% CIs. Linearity of associations between predictors and outcome was assessed by comparing models containing the predictor with natural cubic splines (two, three and four df) with a model containing the raw predictor using likelihood ratio tests. If a variable was non-linear, the optimal number of df was chosen based on statistical significance and marginal effect plots. If NfL was associated with an outcome, we proceeded with multivariable logistic regression analyses for further investigation.

Multivariable logistic regression analyses were employed to assess model performance of clinical prognostic models before and after addition of sNfL or cNfL. In each model, the individual components of the investigated clinical prognostic model were used. Analyses on sNfL or cNfL at a given time point were performed on imputed data from subcohorts containing only patients with an available serum or CSF sample at that given time point. Model performance was evaluated through the C-statistic (discrimination) and R^2^ (explained variance) along with their 95% CI. Correction for optimism was achieved by performing bootstrapping procedures (n=1000).^24^ The added value of a variable was assessed by comparing models before and after the addition of NfL using likelihood ratio tests. If the addition of NfL improved model performance, the model was subsequently compared with simplified models in which one or two clinical factors were removed.

R statistical software (V.4.4.0) packages that were used for analyses include ‘mice’, ‘stats’, ‘pROC’, ‘splines’, ‘auctestr’, ‘rqlm’ and ‘rms’. Two-sided p values <0.05 were considered statistically significant. Bonferroni corrections were applied for multiple comparisons.

### Study data and analytical code availability

Study data and analytical codes used for this study may be made available on reasonable request.

## Results

### Study population and sample availability

**Figure 1:**
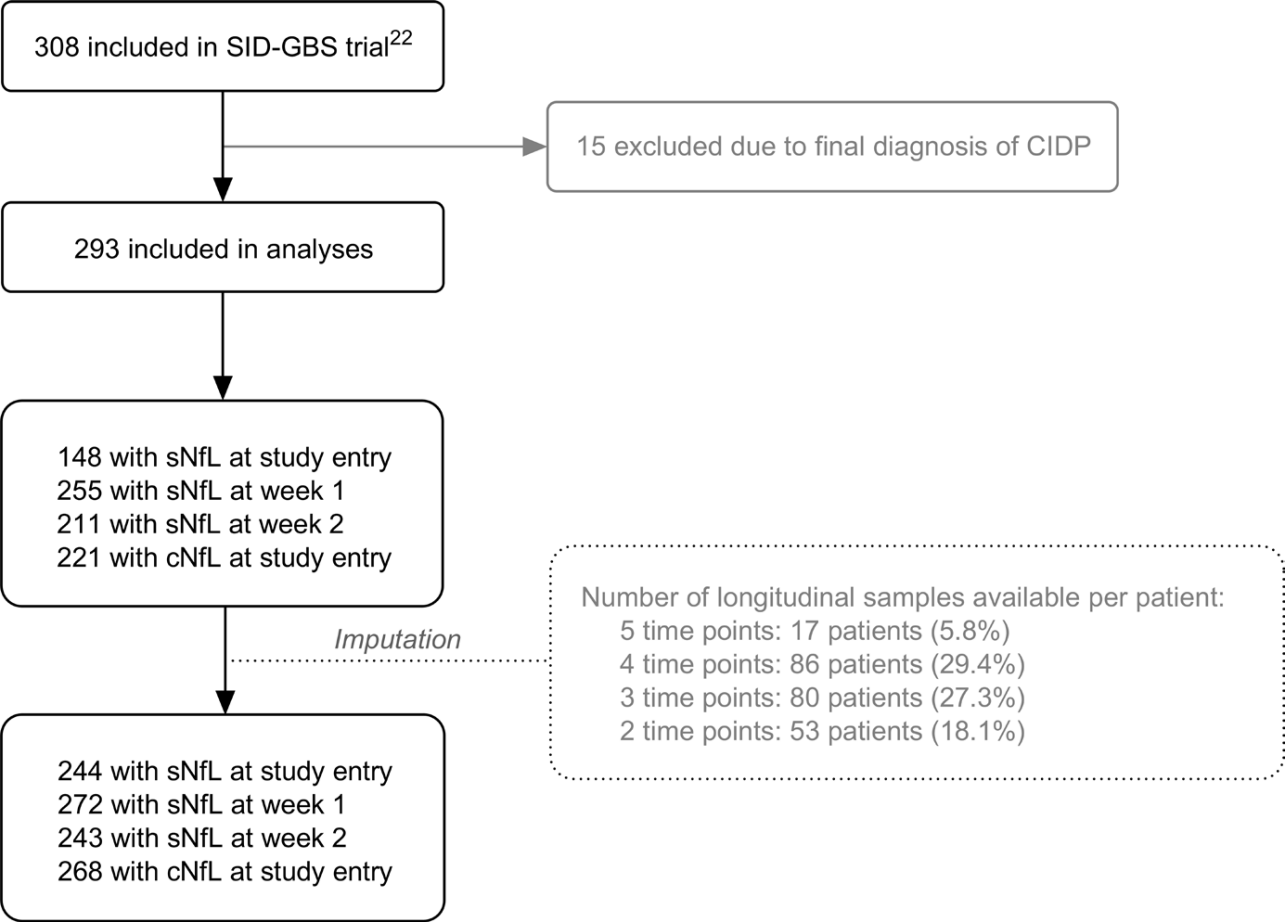
Flow chart for the inclusion and exclusion of patients in this study. For this study, patients included in the randomised placebo-controlled trial with a final diagnosis of chronic inflammatory demyelinating polyneuropathy were excluded. Neurofilament light chain levels at study entry, week 1 and week 2 were imputed if at least two longitudinal samples were available from a given patient. SID-GBS, second intravenous immunoglobulin dose in patients with Guillain-Barré syndrome with poor prognosis; CIDP, chronic inflammatory demyelinating polyneuropathy; (s/c)NfL, (serum/cerebrospinal fluid) neurofilament light chain.^22^

In total, 293 patients with GBS were included in this study (figure 1). Patients had a median age of 57 (IQR: 42–67), and 65.2% of patients were male. A median of 3 (IQR: 2–4) longitudinal serum samples were available per patient. Baseline characteristics did not differ between the SID-GBS cohort and subcohorts based on availability of serum samples at each time point (table 1). Of the 293 included patients, 81 were randomised (43 for placebo, 48 for a second IVIg dose).

**Table 1:** Baseline characteristics of all included patients with Guillain-Barré syndrome and subcohorts based on the availability of serum and cerebrospinal fluid samples at a given time point

	Cohort				
	All (n=293)	sNfL at entry (n=148)	sNfL at week 1 (n=255)	sNfL at week 2 (n=211)	cNfL at entry (n=221)
Demographics					
Age	57 (42–67)	56 (45–65)	57 (44–67)	57 (44–68)	58 (42–67)
Sex (male)	191 (65.2)	99 (66.9)	164 (64.3)	141 (66.8)	139 (62.9)
Clinical features					
Days between onset weakness and admission	2 (1–4)	3 (1–4)	2 (1–4)	2 (1–4)	2 (1–4)
Preceding diarrhoea	82 (28.0)	40 (27.0)	73 (28.6)	62 (29.4)	63 (28.5)
MRC sum score at entry	48 (44–52)	48 (44–52)	48 (44–52)	48 (44–52)	48 (44–52)
MRC sum score at week 1	48 (37–55)	49 (38–56)	49 (38–55)	48 (33–55)	48 (38–54)
MRC score for neck flexion at entry	5 (4–5)	5 (4–5)	5 (4–5)	5 (4–5)	5 (4–5)
MRC score for bilateral hip flexion at entry	8 (6–8)	8 (6–8)	8 (6–8)	8 (6–8)	8 (6–8)
GBS disability score at week 2	3 (2–4)	3 (2–4)	3 (2–4)	3 (2–4)	3 (2–4)
Facial weakness at entry	72 (24.6)	32 (21.6)	61 (23.9)	52 (24.6)	54 (24.4)
Bulbar weakness at entry	51 (17.4)	27 (18.2)	46 (18.0)	44 (20.9)	40 (18.1)
**Outcomes**					
Mechanical ventilation	74 (25.3)	26 (17.6)	63 (24.7)	61 (28.9)	55 (24.9)
Ability to walk 10 m unaided at week 4	133/276 (48.2)	72/139 (51.8)	120/242 (49.6)	93/206 (45.1)	100/212 (47.2)
Ability to walk 10 m unaided at week 26	237/275 (86.2)	122/138 (88.4)	212/242 (87.6)	173/200 (86.5)	184/211 (87.2)
NfL levels					
sNfL at entry (pg/mL)	26 (16–57)	26 (16–57)	26 (16–56)	25 (15–56)	26 (16–57)
sNfL at week 1 (pg/mL)	98 (44–302)	92 (35–250)	98 (44–302)	102 (46–335)	92 (41–281)
sNfL at week 2 (pg/mL)	199 (71–552)	179 (40–497)	200 (73–559)	199 (71–552)	177 (70–508)
cNfL (pg/mL)	585 (330–1062)	594 (358–1079)	578 (337–1049)	613 (347–1075)	585 (330–1062)

Analyses were performed on non-imputed data. Values are presented as n (%) or median (IQR).

GBS, Guillain-Barré syndrome; MRC, Medical Research Council; (s/c)NfL, (serum/cerebrospinal fluid) neurofilament light chain.

Levels of sNfL were available for 148 patients at study entry (additional 96 imputed), 255 at week 1 (additional 17 imputed) and 211 at week 2 (additional 32 imputed), and cNfL at study entry was available for 221 patients (additional 47 imputed; online supplemental figure 1). Imputed NfL levels did not differ from observed NfL levels. Patients without an available serum sample at study entry were admitted faster after onset of weakness and were mechanically ventilated more often than patients with an available sample at study entry, while patients without an available serum sample at week 1 were admitted later after onset of weakness and patients without an available serum sample at week 2 less often had bulbar involvement and less often were mechanically ventilated (online supplemental table 1). Levels of sNfL at week 1, 2, 4 and 12 and cNfL at entry differed between treatment groups (non-randomised, placebo, second IVIg dose), though pairwise comparisons only revealed differences between the non-randomised group and the randomised groups but not between the placebo and second IVIg dose groups; online supplemental figure 2A. No differences in NfL levels were observed between patients with or without additional methylprednisolone treatment (online supplemental figure 2B).

### NfL varies broadly and correlates with predicted outcome

The sNfL levels varied broadly across patients, ranging from 2 pg/mL to 6331 pg/mL at study entry, from 3 pg/mL to 8792 pg/mL at week 1, from 3 pg/mL to 9882 pg/mL at week 2, from 4 pg/mL to 6369 pg/mL at week 4 and from 4 pg/mL to 146 pg/mL at week 12 (median and IQR listed in table 1). At study entry, median cNfL was approximately 22-fold higher than median sNfL (585 (IQR: 330–1062) vs 26 (IQR: 16–57) pg/mL), and log-transformed cNfL and sNfL levels were correlated (online supplemental figure 3). The cNfL:sNfL ratio at study entry ranged from 0.20 to 510.80 (median: 21.75, IQR: 13.16–38.99).

**Figure 2:**
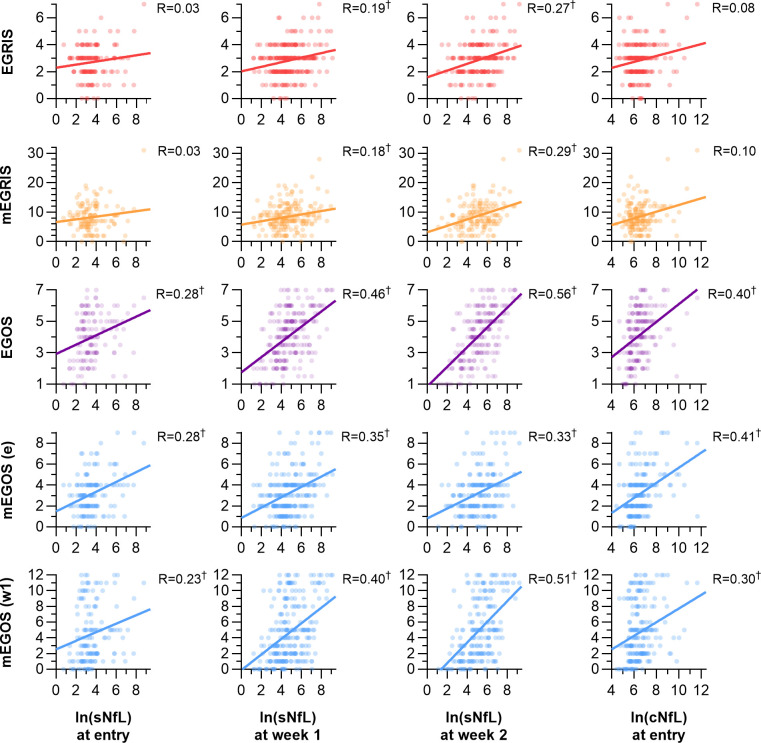
Correlations between scores on clinical prognostic models and NfL at different time points. Each dot represents an individual patient, and each line represents a simple linear regression equation for the given data. NfL levels were log-transformed (natural log). The Spearman correlation coefficient is described as R. Values between 0 and 1 indicate a positive correlation, while values between −1 and 0 indicate a negative correlation. (m)EGOS, (modified) Erasmus Guillain-Barré syndrome Outcome Score; (m)EGRIS, (modified) Erasmus Guillain-Barré syndrome Respiratory Insufficiency Score; (s/c)NfL: (serum/cerebrospinal fluid) neurofilament light chain. ^†^p<0.05.

**Figure 3:**
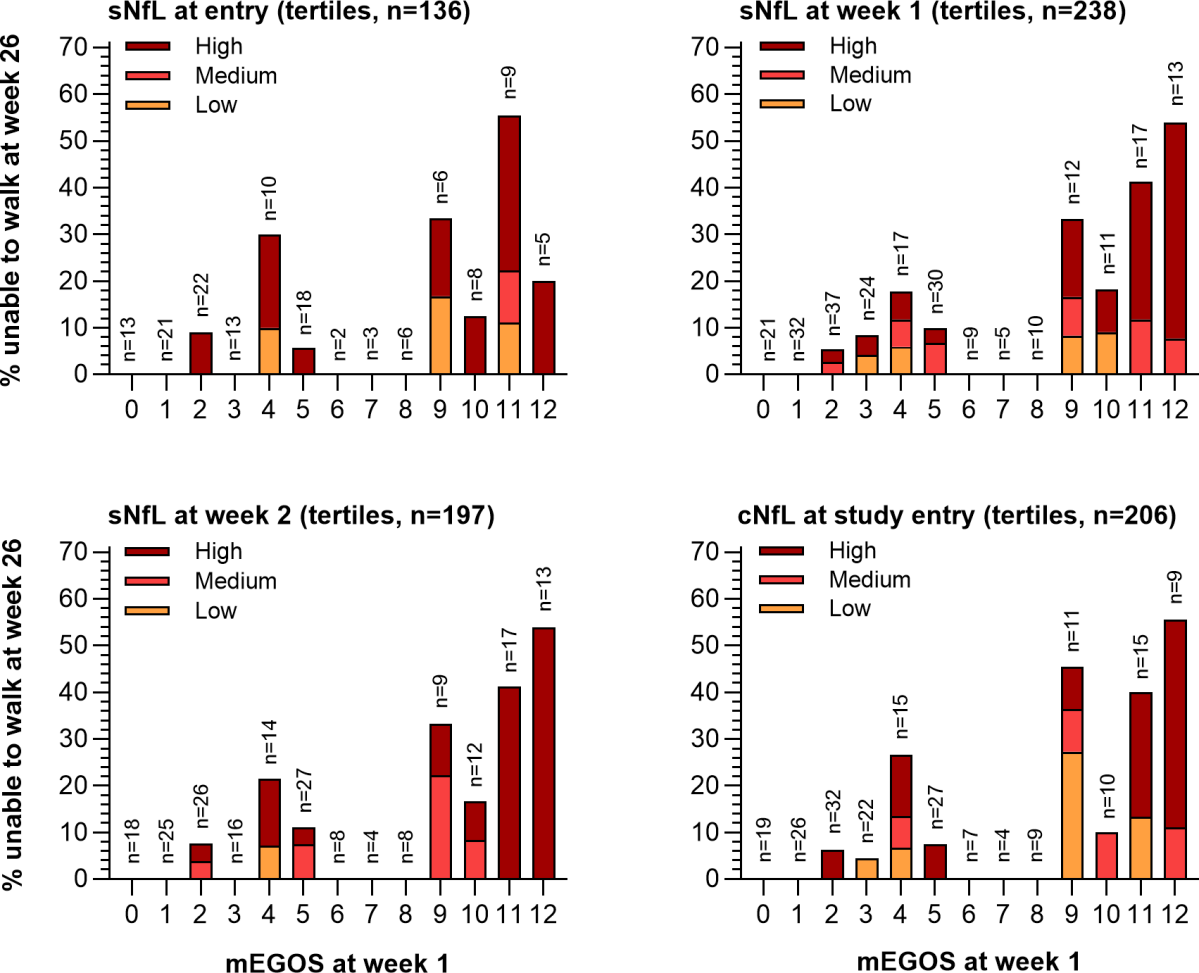
Stacked bar plots for the proportion of patients with a high, medium or low NfL level based on tertiles among patients unable to walk unassisted after 26 weeks of follow-up with a given score on the mEGOS. Patients unable to walk unassisted occur throughout both high and low scores on the mEGOS. A large proportion of these patients have a high (or medium) NfL level. The distinction of a high, medium or low NfL level was made based on tertiles at a given time point. mEGOS, modified Erasmus Guillain-Barré syndrome Outcome Score; (s/c)NfL: (serum/cerebrospinal fluid) neurofilament light chain.

Both log-transformed cNfL and sNfL correlated with individual scores extracted from clinical prognostic models predicting inability to walk (figure 2). Scores on the EGRIS and mEGRIS only correlated with log-transformed sNfL at weeks 1 and 2. Correlations were strongest for sNfL at week 2 with scores on the EGOS and the mEGOS at week 1 (R=0.56 (95% CI 0.45 to 0.65) and R=0.51 (95% CI 0.39 to 0.60)). Notably, high NfL was observed in the majority of patients unable to walk at 26 weeks independent of the mEGOS score at week 1, especially at study entry (figure 3).

### NfL is associated with clinical outcomes

In univariable analyses on the complete SID-GBS cohort, higher sNfL and cNfL at study entry and higher sNfL at week 1 and 2 were associated with the inability to walk at week 4 and 26 (table 2). Generally, these associations were stronger for sNfL than cNfL. Most clinical prognostic factors included in the prognostic models were also associated with the respective outcome. The association of higher sNfL at week 1 and the inability to walk at 26 weeks was non-linear and was best fit using natural cubic splines with two df (online supplemental figure 4). Neither sNfL nor cNfL levels at study entry were associated with respiratory failure in the first week after admission, though sNfL levels at weeks 1 and 2 were associated. Consequently, the addition of NfL to the (m)EGRIS was not explored.

**Table 2:** Univariable logistic regression analyses of (modified) Erasmus Guillain-Barré syndrome Respiratory Insufficiency Score predictors, (modified) Erasmus Guillain-Barré syndrome Outcome Score predictors and NfL with mechanical ventilation and the inability to walk at 4 and 26 weeks of follow-up

	Mechanical ventilation		OR (95% CI)	RR (95% CI)
	No (n=219)	Yes (n=74)		
Days from onset of weakness to admission	3 (1–4)	1 (0–2)	0.77 (0.66 to 0.88)*	0.82 (0.72 to 0.92)*
Facial or bulbar weakness at entry	62 (28.3)	34 (45.9)	2.15 (1.25 to 3.71)*	1.74 (1.18 to 2.57)*
MRC sum score at entry	48 (46–52)	46 (36–51)	0.94 (0.91 to 0.97)*	0.97 (0.96 to 0.98)*
Bulbar weakness at entry	26 (11.9)	25 (33.8)	3.79 (2.01 to 7.16)*	2.42 (1.66 to 3.52)*
MRC score for neck flexion at entry	5 (5–5)	4 (4-5)	0.29 (0.16 to 0.47)*	0.59 (0.51 to 0.69)*
MRC score for bilateral hip flexion at entry	8 (7-8)	8 (6-8)	0.76 (0.66 to 0.87)*	0.84 (0.79 to 0.90)*
sNfL at entry	27 (16–59)	25 (16–53)	0.99 (0.71 to 1.32)	0.99 (0.78 to 1.25)
cNfL at entry	554 (330–1139)	669 (336–953)	0.94 (0.71 to 1.23)	0.96 (0.78 to 1.18)

	Ability to walk 10 m unaided at week 4		OR (95% CI)	RR (95% CI)
	Able (n=133)	Unable (n=143)		
Age	51 (37–62)	61 (48–71)	1.03 (1.01 to 1.04)*	1.01 (1.01 to 1.02)*
Preceding diarrhoea	31 (23.3)	48 (33.6)	1.66 (0.98 to 2.85)	1.26 (1.00 to 1.58)
MRC sum score at entry	50 (47–54)	47 (40–51)	0.93 (0.89 to 0.96)*	0.98 (0.97 to 0.98)*
MRC sum score at week 1	54 (49–57)	38 (19–48)	0.88 (0.84 to 0.91)*	0.97 (0.97 to 0.98)*
sNfL at entry	21 (12–34)	40 (20–106)	1.69 (1.27 to 2.34)*	1.22 (1.11 to 1.34)*
sNfL at week 1	66 (32–199)	160 (83–570)	1.73 (1.42 to 2.14)*	1.25 (1.17 to 1.34)*
sNfL at week 2	89 (30–204)	423 (155–778)	2.39 (1.85 to 3.18)*	1.35 (1.25 to 1.46)*
cNfL at entry	487 (308–832)	718 (409–1283)	1.52 (1.16 to 2.05)*	1.17 (1.07 to 1.27)*

	Ability to walk 10 m unaided at week 26		OR (95% CI)	RR (95% CI)
	Able (n=237)	Unable (n=38)		
Age	56 (41–67)	63 (54–71)	1.03 (1.01 to 1.05)*	1.02 (1.01 to 1.04)*
Preceding diarrhoea	60 (25.3)	16 (42.1)	2.15 (1.04 to 4.34)*	1.90 (1.06 to 3.43)*
GBS disability score at week 2	3 (2–4)	5 (4–5)	4.06 (2.55 to 7.11)*	3.08 (2.34 to 4.06)*
MRC sum score at entry	48 (45–52)	46 (33–51)	0.94 (0.90 to 0.97)*	0.96 (0.94 to 0.97)*
MRC sum score at week 1	50 (42–56)	23 (0–43)	0.93 (0.91 to 0.95)*	0.95 (0.94 to 0.96)*
sNfL at entry	25 (15–48)	96 (45–543)	1.88 (1.35 to 2.70)*	1.60 (1.32 to 1.94)*
sNfL at week 1	87 (41–223)	843 (102–2432)	2.12 (1.61 to 2.88)*†	1.77 (1.44 to 2.17)*†
sNfL at week 2	151 (60–428)	930 (390–2771)	2.73 (1.90 to 4.18)*	2.02 (1.63 to 2.52)*
cNfL at entry	553 (330–1005)	944 (439–5209)	1.75 (1.31 to 2.39)*	1.50 (1.29 to 1.75)*

Analyses were performed on non-imputed data from all included patients. Values are presented as n (%) or median (IQR). NfL levels are presented as raw values, while ORs and RRs were calculated based on the log-transformed values for these variables.

* p<0.05.

† Linear OR.

GBS, Guillain-Barré syndrome; MRC, Medical Research Council; RR, risk ratio; (s/c)NfL, (serum/cerebrospinal fluid) neurofilament light chain.

### Clinical prognostic models improve after adding NfL

**Figure 4:**
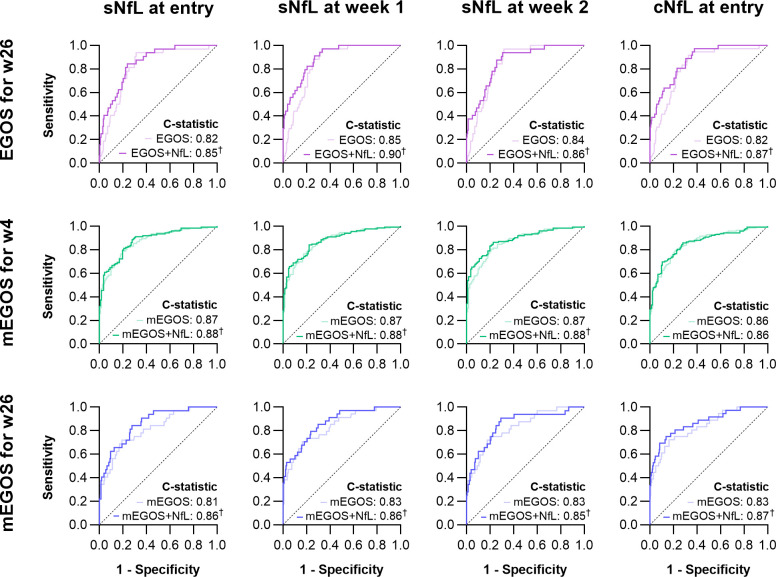
Receiver operating characteristic curves for the mEGOS before and after the addition of NfL. Receiver operating characteristic curves for the mEGOS are shown before (light line) and after (dark line) the addition of NfL. The calculated C-statistic, or area under the receiver operating characteristic curve, is described for each model. (m)EGOS, (modified) Erasmus Guillain-Barré syndrome Outcome Score; (s/c)NfL, (serum/cerebrospinal fluid) neurofilament light chain. ^†^p<0.05.

In multivariable analyses on the subcohorts for complete case analyses, the addition of sNfL and cNfL at each time point increased the C-statistics of the EGOS and the mEGOS (figure 4). Across the subcohorts, the C-statistics for the EGOS (0.82–0.85), the mEGOS at study entry (for week 4: 0.69–0.71; for week 26: 0.67–0.71) and the mEGOS at week 1 (for week 4: 0.86–0.87; for week 26: 0.81–0.83) slightly varied. The range of improvement in C-statistic following the addition of NfL across all models and subcohorts was 0.01–0.11. Generally, the addition of NfL resulted in higher increases in the C-statistic for models predicting outcome at week 26 compared with models predicting outcome at week 4. The highest increase in C-statistic was observed following the addition of sNfL at week 2 to the mEGOS at study entry. However, since the mEGOS generally performed better at week 1 than at study entry, further analyses on the mEGOS were focused on the former. The addition of sNfL at week 1 most prominently increased the C-statistic of the EGOS, and the addition of sNfL at study entry most prominently improved the mEGOS at week 1. Comparison of clinical prognostic models before and after the addition of NfL using likelihood ratio tests showed that the addition of NfL improved model performance for most of the investigated prognostic models, except for cNfL at study entry to the mEGOS predicting outcome for week 4.

Correction of C-statistics for optimism using bootstrapping (n=1000) resulted in slightly decreased C-statistics, though the ∆C-statistics remained constant (table 3). Replication of these analyses with non-imputed raw data and Z-transformed (age-standardised) data in the non-imputed dataset resulted in similar findings (online supplemental tables 2–4). Though a combination of sNfL and cNfL at study entry further improved prognostic models for the inability to walk at 26 weeks based on imputed data (optimism-corrected C-statistics: 0.87 (EGOS) and 0.85 (mEGOS at week 1)), this could not be replicated in non-imputed data.

**Table 3:** Model performance statistics, including statistics for individual model components, for the models that improved most prominently following the addition of neurofilament light chain

Model components	Erasmus GBS Outcome Score (prediction for week 26)			
	OR (95% CI)		RR (95% CI)	
	Model	Model+NfL	Model	Model+NfL
Age	1.01 (0.99 to 1.04)	1.01 (0.98 to 1.04)	1.01 (0.99 to 1.03)	1.01 (0.98–1.03)
Preceding diarrhoea	1.61 (0.68 to 3.75)	1.03 (0.39 to 2.59)	1.37 (0.73 to 2.55)	0.97 (0.56–1.68)
GBS disability score at week 2	3.57 (2.25 to 6.22)*	3.06 (1.90 to 5.43)*	2.84 (2.15 to 3.75)*	2.40 (1.81–3.19)*
Log sNfL at week 1		1.82 (1.33 to 2.56)*†		1.42 (1.17–1.73)*†
	**C-statistic (95% CI)‡**		**R**^**2**^ **(95% CI)‡**	
	**Model**	**Model+NfL**	**Model**	**Model+NfL**
	0.84 (0.77 to 0.90)	0.88 (0.82 to 0.94)*	0.30 (0.19 to 0.46)	0.44 (0.29–0.62)

Model components	Modified Erasmus GBS Outcome Score (prediction for week 4)			
	OR (95% CI)		RR (95% CI)	
	Model	Model+NfL	Model	Model+NfL
Age	1.03 (1.01 to 1.05)*	1.02 (1.00 to 1.05)*	1.01 (1.00 to 1.02)*	1.01 (1.00–1.02)*
Preceding diarrhoea	1.06 (0.51 to 2.19)	0.91 (0.42 to 1.93)	1.01 (0.80 to 1.26)	0.98 (0.78–1.23)
MRC sum score at week 1	0.87 (0.83 to 0.91)*	0.87 (0.83 to 0.91)*	0.98 (0.97 to 0.98)*	0.98 (0.97–0.98)*
Log sNfL at study entry		1.36 (1.11 to 1.69)*		1.05 (1.00–1.11)
	**C-statistic (95% CI)‡**		**R**^**2**^ **(95% CI)‡**	
	**Model**	**Model+NfL**	**Model**	**Model+NfL**
	0.86 (0.80 to 0.91)	0.87 (0.82 to 0.92)*	0.48 (0.36 to 0.63)	0.51 (0.38–0.66)

Model components	Modified Erasmus GBS Outcome Score (prediction for week 26)			
	(95% CI)		RR (95% CI)	
	Model	Model+NfL	Model	Model+NfL
Age	1.03 (1.00 to 1.06)	1.02 (0.99 to 1.06)	1.02 (1.00 to 1.04)	1.02 (0.99–1.04)
Preceding diarrhoea	1.18 (0.45 to 2.91)	0.93 (0.34 to 2.40)	1.04 (0.58 to 1.89)	0.88 (0.47–1.64)
MRC sum score at week 1	0.94 (0.92 to 0.96)*	0.94 (0.91 to 0.96)*	0.96 (0.94 to 0.97)*	0.96 (0.95–0.98)*
Log sNfL at study entry		1.51 (1.20 to 1.94)*		1.24 (1.07–1.45)*
	**C-statistic (95% CI)** **‡**		**R**^**2**^ **(95% CI)****‡**	
	**Model**	**Model+NfL**	**Model**	**Model+NfL**
	0.80 (0.69 to 0.90)	0.84 (0.76 to 0.92)*	0.28 (0.11 to 0.49)	0.35 (0.18–0.56)

Analyses were performed on the imputed datasets of subcohorts based on the availability of neurofilament light chain levels at a given time point.

* p<0.05.

† Linear odds ratio.

‡ Optimism-corrected.

GBS, Guillain-Barré syndrome; MRC, Medical Research Council; RR, risk ratio; (s)NfL, (serum) neurofilament light chain.

### Combining NfL with disease severity measures has the highest prognostic value

Following stepwise removal of clinical predictors from the mEGOS at week 1 with sNfL at study entry predicting outcome at week 26, optimism-corrected C-statistics remained comparable to the initial model (table 4). Similar patterns were found for other prognostic models (online supplemental table 5). The MRC sum score and GBS-DS consistently were the strongest prognostic factors alongside NfL (table 4 and online supplemental table 5). Combining these three factors into a new prognostic model predicting the inability to walk at 26 weeks resulted in the best model performance of all investigated models containing sNfL at study entry (optimism-corrected C-statistic: 0.86 (95% CI 0.78 to 0.93)). When adjusting the MRC sum score to week 2 and the GBS-DS to study entry alongside sNfL at study entry, model performance improved even further (optimism-corrected C-statistic: 0.88 (95% CI 0.83 to 0.94)). Similarly, combining sNfL at study entry with the MRC sum score at week 1 and the GBS-DS at week 1 for outcome prediction at week 4 also resulted in the highest model performance of all models containing sNfL at study entry (optimism-corrected C-statistic: 0.91 (95% CI 0.87 to 0.96)).

**Table 4:** Model performance statistics for the mEGOS with serum NfL added before and after the removal of clinical predictors

	C-statistic* (95% CI)	R^2^ (95% CI)
mEGOS at week 1+sNfL at entry (prediction for week 26)		
Age+preceding diarrhoea+MRC sum score at week 1+sNfL at entry	0.84 (0.76 to 0.92)	0.35 (0.18 to 0.56)
Removal of one clinical prognostic factor		
Age+MRC sum score at week 1+sNfL at entry	0.85 (0.76 to 0.93)†	0.36 (0.18 to 0.56)†
Preceding diarrhoea+MRC sum score at week 1+sNfL at entry	0.84 (0.76 to 0.92)†	0.35 (0.16 to 0.55)†
Age+preceding diarrhoea+sNfL at entry	0.73 (0.61 to 0.86)	0.15 (0.01 to 0.37)
Removal of two clinical prognostic factors		
MRC sum score at week 1+sNfL at entry	0.85 (0.76 to 0.92)†	0.36 (0.17 to 0.55)†
Age+sNfL at entry	0.74 (0.61 to 0.86)	0.17 (0.02 to 0.37)
Preceding diarrhoea+sNfL at entry	0.72 (0.58 to 0.85)	0.15 (0.02 to 0.34)

Analyses were performed in the imputed dataset of patients with available serum neurofilament light chain levels at entry.

* Optimism-corrected.

† Performs equally to the full model with neurofilament light chain added.

mEGOS, modified Erasmus Guillain-Barré syndrome Outcome Score; MRC, Medical Research Council; (s)NfL, (serum) neurofilament light chain.

## Discussion

In this study, we showed that, in patients with GBS, the addition of sNfL or cNfL improves the performance of clinical prognostic models predicting inability to walk but not of models predicting respiratory failure. In addition, NfL has the potential to replace specific clinical scores in prognostic models, such as preceding diarrhoea, and a model containing sNfL, the MRC sum score and the GBS-DS may perform best. The concordance between clinical factors and NfL may provide more certainty for clinical evaluations and decisions.

Our findings are in line with previous studies on NfL as prognostic biomarkers. In GBS, the prognostic value of NfL has been investigated in several studies.^14^,^21^ Similar to our findings, these studies described associations of NfL with the disease course and outcomes. In our study, we were able to build on existing evidence by performing further analyses on the added prognostic value of NfL to clinical prognostic models in a well-defined cohort of patients with GBS. The prognostic value of NfL has also been extensively studied in a plethora of other neurological conditions, including multiple sclerosis, dementia and traumatic brain injury.^13^ In these studies, sNfL and cNfL have also been repeatedly associated with the disease course and outcomes. Some studies on other neurological diseases have also determined incremental prognostic value of NfL to clinical prognostic models, as in traumatic brain injury.^25^

Associations of sNfL and cNfL with the disease course and outcomes in GBS are most likely explained by damage to axons in which NfL is exclusively expressed.^13^ NfL may be released in the serum after axonal damage of peripheral nerves, which may be either primary, secondary or a combination of both. In addition, NfL in CSF may be released as a consequence of ischaemic damage to proximal nerve trunks/roots after inflammatory oedema.^26^ Patients with more extensive acute-phase axonal damage, and thus high sNfL and cNfL, often present with more severe disease and recover poorly.^3 4 9^ Axonal damage is especially relevant for the long-term outcome of GBS, since regeneration of myelin generally occurs faster than of axons.^27^ This mechanism may explain the observed high predictive value of high NfL levels for the occurrence of poor recovery after 26 weeks in our study. In contrast, in the acute stage of GBS, other mechanisms than axonal degeneration may determine disease severity and the occurrence of respiratory failure.

In the very early stages of GBS, ischaemic proximal nerve damage and demyelination have been shown to be important pathological features.^28^ Both of these mechanisms may cause conduction block, which has been implicated as a potential cause of respiratory failure.^28^,^30^ In addition, some studies have specifically shown that patients with demyelinating pathology have an increased risk of developing respiratory failure.^30 31^ The contribution of these disease mechanisms to the development of respiratory failure may explain why NfL at study entry was not associated with this outcome. Also, though some axonal damage to respiratory nerves may occur and contribute to the development of respiratory failure, the NfL levels determined at study entry probably largely reflect the extent of early axonal damage to longer nerves in the limbs instead. In our study, we found that the majority of patients have higher cNfL than sNfL at study entry, suggesting that a more proximal distribution of pathology with leakage of NfL from CSF to serum may also be the predominant early-stage pathological mechanism in these patients. Moreover, our finding that NfL at entry and acute-phase disease severity measures were both independently associated with the inability to walk in prognostic models further supports the idea that other pathological mechanisms besides axonal damage contribute to early-stage disease severity.

The development of prognostic models including NfL has important implications for clinical practice and future research.^8^ Early prognostication of patients is useful for guiding treatment and care, informing patients and their relatives, selecting patients for clinical trials, conducting observational treatment studies and enabling prognostic stratification of patients. Although clinical prognostic factors are generally easier to obtain, the addition of NfL could be valuable and feasible in clinical practice. First, NfL may be a more objective alternative predictor than some clinical predictors such as preceding diarrhoea, which is often misreported. Likewise, clinical assessment of muscle strength is subject to interobserver variability and can be complicated in settings such as intensive care units.^32 33^ Second, serum and CSF are usually already acquired in the diagnostic work-up, and testing of NfL will be no additional burden for patients. Third, an increasing number of institutions have technology available to determine NfL levels. Though routine testing may currently take up to a week, recent implementation of assays into random-access laboratory technology will provide opportunities to acquire results within a day in the near future. Importantly, acquiring the NfL test result after 1 week will not delay the use of prognostic models, since NfL time points precede clinical prognostic factor time points by a week in each model. Accessible and reliable routine NfL testing is required to implement a model containing NfL in clinical practice.

This study has several limitations. First, the study population was relatively small, resulting in limited statistical power for analyses. Second, since included patients were selected for a clinical trial based on stringent inclusion and exclusion criteria, selection bias may have occurred. Similarly, missing data may also have introduced some degree of selection bias. The limitations related to limited power and selection bias could in part be overcome through data imputation. To further address these limitations, the NfL-based prognostic models should be validated in larger, more heterogeneous and independent patient cohorts before implementation into clinical practice. Third, the different treatment regimens administered in the SID-GBS trial may have affected NfL levels, although we found no differences between the placebo group and the second IVIg dose group. With the assumption that administration of albumin as placebo does not affect NfL levels, bias resulting from different treatment regimens is therefore expected to be limited. Fourth, NfL is released from both central and peripheral nervous system neurons, and thus determination of levels may be confounded by potential (subclinical) comorbidities. For this study, patients with polyneuropathy from other causes and patients with severe concurrent disease were excluded.^22^ Nevertheless, models containing NfL should be interpreted with caution in patients with comorbidities that may affect NfL levels. Biomarkers more specific for peripheral nerve damage, such as peripherin, may be a potential future alternative.^34^ However, several other factors are important for the clinical application of biomarkers, such as stability, accessibility and robustness. Adding a combination of multiple biomarkers, such as markers reflecting myelin integrity or glial fibrillar acidic protein, to clinical prognostic models or creating new prognostic models with biomarkers may even further increase predictive performance.

In conclusion, the addition of sNfL or cNfL improved the model performance of clinical prognostic models predicting inability to walk but not of models predicting respiratory failure in GBS. Moreover, NfL has the potential to replace clinical predictors in prognostic models and may perform best in conjunction with measures of acute-phase disease severity.

## Supplementary material

10.1136/jnnp-2025-336046 online supplemental figure 1

10.1136/jnnp-2025-336046 online supplemental file 1

## Data Availability

Data are available upon reasonable request.
