# Supplementary material for: Neurofilament light chain improves clinical prognostic models for Guillain-Barré syndrome
Source: J Neurol Neurosurg Psychiatry. 2025 May 2;96(11):e336046. doi: 10.1136/jnnp-2025-336046 (PMC12573411; doi:10.1136/jnnp-2025-336046)
Supplement: online supplemental figure 1 [file jnnp-96-11-s001.pdf]

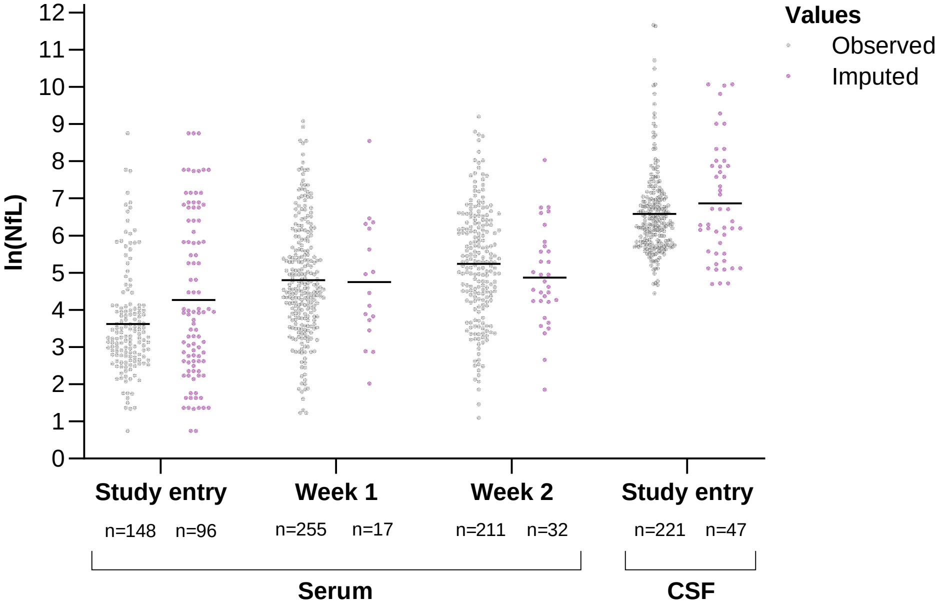

Supplementary Fig. 1: Dot plots for observed and imputed neurofilament light chain levels. Black horizontal lines indicate medians. Imputed ln(NfL) did not differ from observed ln(NfL) for any of the matrices or time points. NfL: neurofilament light chain, CSF: cerebrospinal fluid.

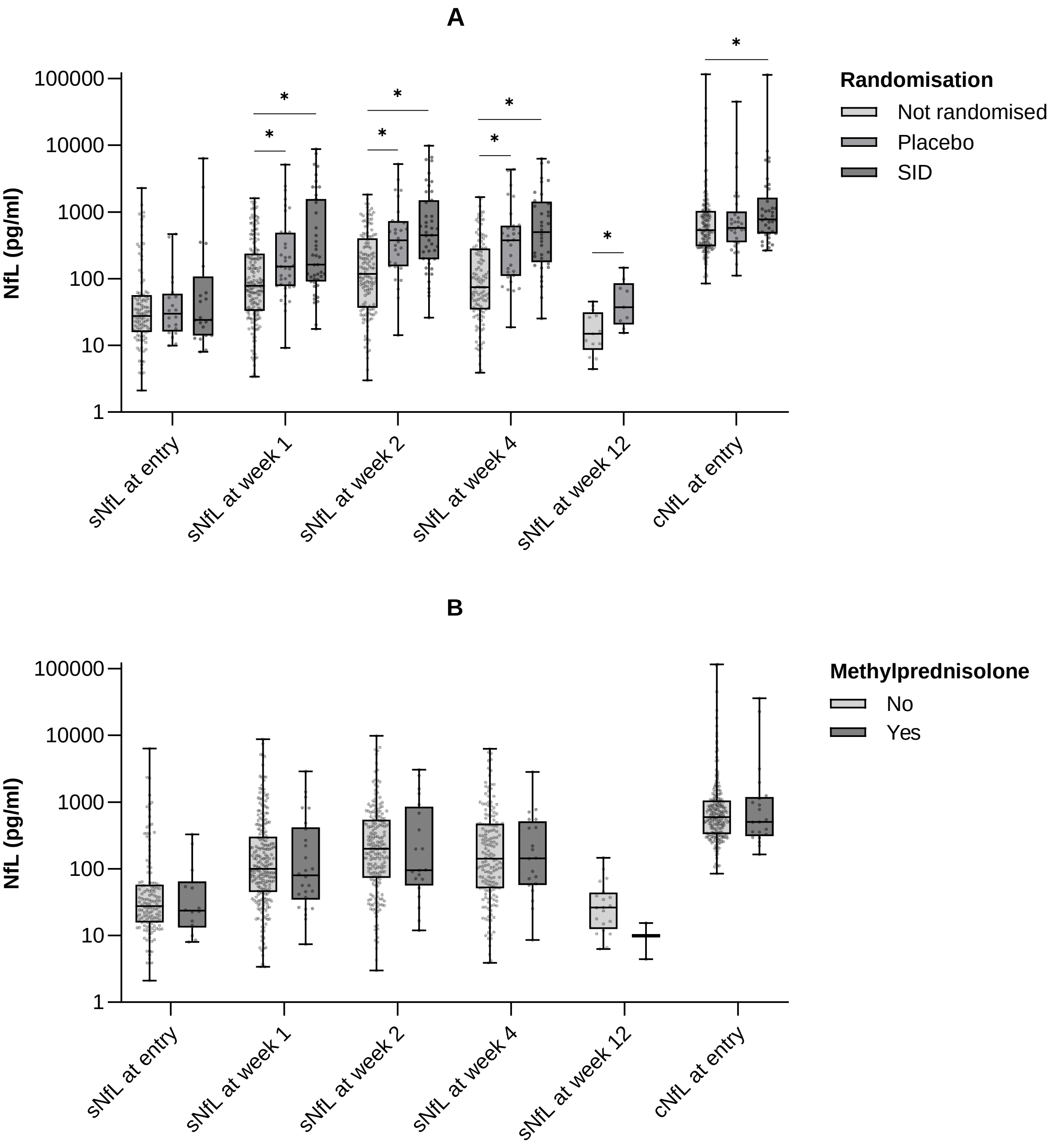

Supplementary Fig. 2: Box plots for neurofilament light chain levels per treatment group. Comparisons are shown for groups based on randomisation procedures (not randomised, placebo, or second IVIg dose; A) as previously described and additional treatment with methylprednisolone (B).[18, 22] Each dot represents an individual patient. (s/c)NfL: serum/cerebrospinal fluid neurofilament light chain, SID: second IVIg dose. * $p < 0.05$.

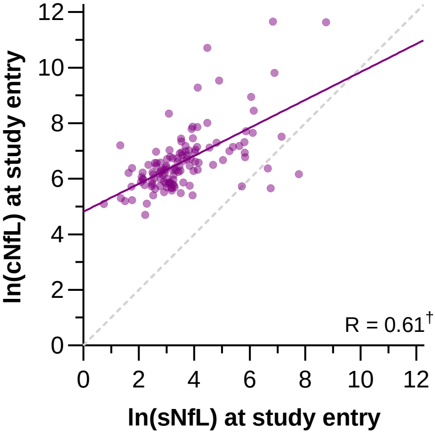

Supplementary Fig. 3: Correlation plot for the correlation between serum and cerebrospinal fluid neurofilament light chain at study entry. Neurofilament light chain levels were log-transformed (natural log). Each dot represents an individual patient. The solid line represents the regression line acquired from linear regression. The dashed line represents a reference line (x=y). The Spearman correlation coefficient was described as R. (s/c)NfL: serum/cerebrospinal fluid neurofilament light chain. † $p<0.05$.

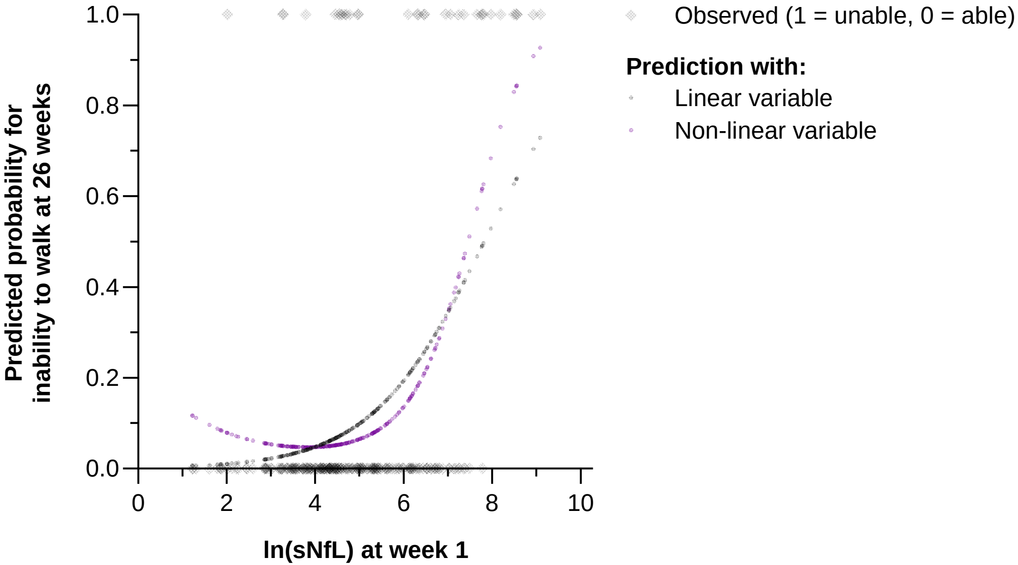

Supplementary Fig. 4: Marginal effect plot for the predicted probabilities of the inability to walk at 26 weeks based on the linear and non-linear variable for serum neurofilament light chain at week 1. Serum neurofilament light chain levels were log-transformed (natural log). sNfL: serum neurofilament light chain.

Supplementary File 1: Procedures applied for imputation of missing data

Using the "mice" package, missing continuous data were imputed through predictive mean matching and missing categorical data through logistic regression. Imputations were redone after our previous study, due to two additional patient exclusions (final diagnosis of chronic inflammatory demyelinating neuropathy) and minor adjustments to neurofilament light chain time point definitions.[1] Single imputation was chosen because the use of one dataset was more suitable for the performed analyses than the use of multiple datasets acquired from multiple imputation.

Missing clinical data were imputed if <15% of data from a given clinical variable was missing. Since none of the analysed clinical variables had ≥15% missing values, all relevant clinical variables could be imputed. Outcomes (mechanical ventilation and inability to walk 10 meters unaided at week 4 and week 26) were not imputed to avoid bias and overfitting. Missing neurofilament light chain data from entry (serum and cerebrospinal fluid), week 1 (only serum), and week 2 (only serum) were imputed for patients who had available neurofilament light chain data from at least two time points.

Input variables were determined based on clinical relevance and included age, sex, preceding diarrhoea, Guillain-Barré syndrome disability scores (entry, week 1, week 2, week 4, week 8, week 12, and week 26), Medical Research Council (sum) scores (entry, week 1, week 2, week 4, week 8, week 12, and week 26), cranial nerve palsy (facial, bulbar, and oculomotor; entry, week 1, week 2, week 4, week 8, week 12, and week 26), number of days between onset of weakness and hospital admission, electrophysiological subtypes as classified by Hadden, inability to walk unaided (week 4 and week 26), mechanical ventilation, and neurofilament light chain levels (serum [entry, week 1, week 2, week 4, and week 12] and cerebrospinal fluid [entry]).[2]

References

1. van Tilburg SJ, Teunissen CE, Maas CCHM, et al. Dynamics and prognostic value of serum neurofilament light chain in Guillain-Barré syndrome. EBioMedicine 2024;102:105072.
2. Hadden RD, Cornblath DR, Hughes RA, et al. Electrophysiological classification of Guillain-Barré syndrome: clinical associations and outcome. Plasma Exchange/Sandoglobulin Guillain-Barré Syndrome Trial Group. Ann Neurol 1998;44(5):780-788.

Supplementary Table 1: Comparison of clinical features between patients with or without an available serum or cerebrospinal fluid sample at each time point.

	**Serum sample availability at entry**		**Serum sample availability at week 1**		**Serum sample availability at week 2**		**CSF sample availability at entry**	
**Variable**	**Available (n=148)**	**Missing (n=145)**	**Available (n=255)**	**Missing (n=38)**	**Available (n=211)**	**Missing (n=82)**	**Available (n=221)**	**Missing (n=72)**
**Demographics**								
Age	56 (45-65)	58 (40-68)	57 (44-67)	51 (33-63)	57 (44-68)	55 (37-66)	58 (42-67)	54 (41-65)
Sex (female)	49 (33.1)	53 (36.6)	91 (35.7)	11 (28.9)	70 (33.2)	32 (39.0)	82 (37.1)	20 (27.8)
**Clinical features**								
Days between onset weakness and admission	3 (1-4)†	2 (1-4)†	2 (1-4)†	4 (2-4)†	2 (1-4)	3 (1-4)	2 (1-4)	2 (1-4)
Preceding diarrhoea	40 (27.0)	42 (29.0)	73 (28.6)	9 (23.7)	62 (29.4)	20 (24.4)	63 (28.5)	19 (26.4)
MRC sum score at entry	48 (44-52)	48 (44-52)	48 (44-52)	49 (43-52)	48 (44-52)	50 (45-54)	48 (44-52)	48 (45-51)
MRC sum score at week 1	49 (38-56)	48 (33-54)	49 (38-55)	48 (27-51)	48 (33-55)	50 (44-54)	48 (38-54)	50 (33-55)
MRC score for neck flexion at entry	5 (4-5)	5 (4-5)	5 (4-5)	5 (4-5)	5 (4-5)	5 (4-5)	5 (4-5)	5 (4-5)
MRC score for bilateral hip flexion at entry	8 (6-8)	8 (6-8)	8 (6-8)	8 (8-8)	8 (6-8)	8 (7-8)	8 (6-8)	8 (6-8)
GBS disability score at week 2	3 (2-4)	3 (2-5)	3 (2-4)	3 (3-5)	3 (2-4)	3 (2-4)	3 (2-4)	3 (2-4)
Facial weakness at entry	32 (21.6)	40 (145)	61 (23.9)	11 (28.9)	52 (24.6)	20 (24.4)	54 (24.4)	18 (25.0)
Bulbar weakness at entry	27 (18.2)	24 (16.6)	46 (18.0)	5 (13.2)	44 (20.9)†	7 (8.5)†	40 (18.1)	11 (15.3)
**Outcomes**								
Mechanical ventilation	26 (17.6)†	48 (33.1)†	63 (24.7)	11 (28.9)	61 (28.9)†	13 (15.9)†	55 (24.9)	19 (26.4)
Unable to walk 10 meters unaided at week 4	67/139 (48.2)	76/137 (55.5)	122/242 (50.4)	21/34 (61.8)	113/206 (54.9)	30/70 (42.9)	112/212 (52.8)	31/64 (48.4)
Unable to walk 10 meters unaided at week 26	16/138 (11.6)	22/137 (16.1)	30/242 (12.4)	8/33 (24.2)	27/200 (13.5)	11/75 (14.7)	27/211 (12.8)	11/64 (17.2)

Data are presented as count (%) or median (interquartile range). Comparisons between patients with or without an available serum sample at week 4 and 12 were not shown, because these data were not imputed.
CSF: cerebrospinal fluid, MRC: Medical Research Council, GBS: Guillain-Barré syndrome.
†$p<0.05$.

Supplementary Table 2: Model performance statistics in the non-imputed dataset, including statistics for individual model components, for the models that improved most prominently following addition of neurofilament light chain.

	**Erasmus GBS Outcome Score (prediction for week 26)**			
	**Odds ratio (95% CI)**		**Risk ratio (95% CI)**	
**Model components**	**Model**	**Model + NfL**	**Model**	**Model + NfL**
Age	1.01 (0.99-1.05)	1.01 (0.98-1.04)	1.01 (0.99-1.03)	1.00 (0.98-1.03)
Preceding diarrhoea	1.49 (0.58-3.73)	1.03 (0.37-2.76)	1.29 (0.65-2.57)	0.98 (0.55-1.74)
GBS disability score at week 2	3.50 (2.14-6.31)[†]	3.02 (1.81-5.58)[†]	2.76 (2.07-3.68)[†]	2.35 (1.73-3.19)[†]
Log sNfL at week 1		1.78 (1.28-2.56)[†,a]		1.40 (1.13-1.74)[†,a]
	**C-statistic (95% CI)***		**R^2 (95% CI)***	
	**Model**	**Model + NfL**	**Model**	**Model + NfL**
	0.83 (0.75-0.91)	0.88 (0.81-0.95)[†]	0.29 (0.15-0.49)	0.45 (0.27-0.67)[†]

	**Modified Erasmus GBS Outcome Score (prediction for week 4)**			
	**Odds ratio (95% CI)**		**Risk ratio (95% CI)**	
**Model components**	**Model**	**Model + NfL**	**Model**	**Model + NfL**
Age	1.03 (1.00-1.06)[†]	1.02 (0.99-1.05)	1.01 (1.00-1.02)[†]	1.01 (1.00-1.02)
Preceding diarrhoea	0.75 (0.29-1.90)	0.62 (0.22-1.63)	0.94 (0.68-1.30)	0.82 (0.58-1.14)
MRC sum score at week 1	0.89 (0.84-0.93)[†]	0.89 (0.84-0.93)[†]	0.97 (0.97-0.98)[†]	0.97 (0.97-0.98)[†]
Log sNfL at study entry		1.75 (1.24-2.60)[†]		1.17 (1.04-1.31)[†]
	**C-statistic (95% CI)***		**R^2 (95% CI)***	
	**Model**	**Model + NfL**	**Model**	**Model + NfL**
	0.82 (0.74-0.91)	0.86 (0.78-0.93)[†]	0.40 (0.23-0.62)	0.46 (0.29-0.69)[†]

	**Modified Erasmus GBS Outcome Score (prediction for week 26)**			
	**Odds ratio (95% CI)**		**Risk ratio (95% CI)**	
**Model components**	**Model**	**Model + NfL**	**Model**	**Model + NfL**
Age	1.02 (0.98-1.07)	1.01 (0.97-1.06)	1.02 (0.98-1.06)	1.02 (0.98-1.06)
Preceding diarrhoea	1.27 (0.34-4.35)	0.69 (0.15-2.70)	1.20 (0.49-2.92)	0.71 (0.27-1.91)
MRC sum score at week 1	0.94 (0.91-0.97)[†]	0.93 (0.89-0.96)[†]	0.95 (0.94-0.97)[†]	0.96 (0.93-0.98)[†]
Log sNfL at study entry		2.06 (1.31-3.45)[†]		1.41 (1.13-1.75)[†]
	**C-statistic (95% CI)***		**R^2 (95% CI)***	
	**Model**	**Model + NfL**	**Model**	**Model + NfL**
	0.76 (0.59-0.94)	0.82 (0.67-0.95)[†]	0.18 (0-0.52)	0.28 (0.05-0.63)[†]

Analyses were performed on the non-imputed datasets of subcohorts based on the availability of serum samples at a given time point.
GBS: Guillain-Barré syndrome, CI: confidence interval, (s)NfL: (serum) neurofilament light chain, MRC: Medical Research Council.
* optimism-corrected, † $p<0.05$, [a] linear odds ratio.

Supplementary Table 3: Univariable analyses for Z-transformed serum neurofilament light chain with mechanical ventilation and the ability to walk 10 meters unaided at 4 weeks and 26 weeks of follow-up.

	Mechanical ventilation		OR (95% CI)	RR (95% CI)
	**No (n=219)**	**Yes (n=74)**		
sNfL at study entry (Z-score)	2.58 (1.32-4.00)	1.91 (1.60-3.05)	0.95 (0.80-1.11)	0.96 (0.83-1.11)
	**Ability to walk 10 meters unaided at 4 weeks**		**OR (95% CI)**	**RR (95% CI)**
	**Able (n=133)**	**Unable (n=143)**		
sNfL at study entry (Z-score)	1.94 (0.59-3.12)	2.80 (1.68-5.24)	1.21 (1.06-1.41)[†]	1.08 (1.03-1.13)[†]
sNfL at week 1 (Z-score)	4.39 (2.69-6.17)	5.98 (4.30-8.30)	1.24 (1.13-1.37)[†]	1.10 (1.06-1.14)[†]
sNfL at week 2 (Z-score)	4.61 (2.79-6.61)	7.65 (5.36-9.17)	1.43 (1.28-1.63)[†]	1.14 (1.10-1.19)[†]
	**Ability to walk 10 meters unaided at 26 weeks**		**OR (95% CI)**	**RR (95% CI)**
	**Able (n=237)**	**Unable (n=38)**		
sNfL at study entry (Z-score)	2.05 (1.32-3.21)	5.10 (2.52-7.86)	1.33 (1.13-1.58)[†]	1.24 (1.13-1.37)[†]
sNfL at week 1 (Z-score)	4.90 (3.19-6.85)	8.21 (4.38-11.31)	1.40 (1.22-1.62)[†,a]	1.30 (1.17-1.45)[†,a]
sNfL at week 2 (Z-score)	5.70 (3.86-7.96)	8.64 (7.37-11.43)	1.51 (1.28-1.82)[†]	1.36 (1.22-1.52)[†]

Analyses were performed on non-imputed data from all included patients. Serum neurofilament light chain levels were transformed into age-standardised Z-scores. Values are presented as n (%) or median (interquartile range).
OR: odds ratio, CI: confidence interval, sNfL: serum neurofilament light chain.
[†] p<0.05, [a] linear odds ratio.

Supplementary Table 4: Model performance statistics in the non-imputed dataset with Z-transformed neurofilament light chain, including statistics for individual model components, for the three models that improved most prominently following addition of neurofilament light chain.

	**Erasmus GBS Outcome Score (prediction for week 26)**			
	**Odds ratio (95% CI)**		**Risk ratio (95% CI)**	
**Model components**	**Model**	**Model + NfL**	**Model**	**Model + NfL**
Age	1.01 (0.99-1.05)	1.02 (0.99-1.06)	1.01 (0.99-1.03)	1.01 (0.99-1.04)
Preceding diarrhoea	1.49 (0.58-3.73)	1.03 (0.37-2.75)	1.29 (0.65-2.57)	0.98 (0.55-1.74)
GBS disability score at week 2	3.50 (2.14-6.31)†	3.04 (1.82-5.62)†	2.76 (2.07-3.68)†	2.36 (1.74-3.20)†
sNfL at week 1 (Z-score)		1.32 (1.13-1.58)†,a		1.18 (1.06-1.31)†,a
	**C-statistic (95% CI)***		**R^2 (95% CI)***	
	**Model**	**Model + NfL**	**Model**	**Model + NfL**
	0.83 (0.75-0.91)	0.87 (0.76-0.94)†	0.29 (0.15-0.49)	0.43 (0.17-0.59)†

	**Modified Erasmus GBS Outcome Score (prediction for week 4)**			
	**Odds ratio (95% CI)**		**Risk ratio (95% CI)**	
**Model components**	**Model**	**Model + NfL**	**Model**	**Model + NfL**
Age	1.03 (1.00-1.06)†	1.03 (1.01-1.06)†	1.01 (1.00-1.02)†	1.01 (1.00-1.03)†
Preceding diarrhoea	0.75 (0.29-1.90)	0.60 (0.22-1.60)	0.94 (0.68-1.30)	0.82 (0.58-1.14)
MRC sum score at week 1	0.89 (0.84-0.93)†	0.89 (0.84-0.93)†	0.97 (0.97-0.98)†	0.97 (0.97-0.98)†
sNfL at entry (Z-score)		1.30 (1.10-1.58)†		1.08 (1.02-1.14)†
	**C-statistic (95% CI)***		**R^2 (95% CI)***	
	**Model**	**Model + NfL**	**Model**	**Model + NfL**
	0.82 (0.74-0.91)	0.85 (0.78-0.93)†	0.40 (0.23-0.62)	0.46 (0.29-0.68)†

	**Modified Erasmus GBS Outcome Score (prediction for week 26)**			
	**Odds ratio (95% CI)**		**Risk ratio (95% CI)**	
**Model components**	**Model**	**Model + NfL**	**Model**	**Model + NfL**
Age	1.02 (0.98-1.07)	1.03 (0.99-1.09)	1.02 (0.98-1.06)	1.03 (0.99-1.07)
Preceding diarrhoea	1.27 (0.34-4.35)	0.69 (0.15-2.69)	1.20 (0.49-2.92)	0.71 (0.27-1.90)
MRC sum score at week 1	0.94 (0.91-0.97)†	0.93 (0.89-0.96)†	0.95 (0.94-0.97)†	0.96 (0.93-0.98)†
sNfL at entry (Z-score)		1.42 (1.14-1.82)†		1.18 (1.06-1.31)†
	**C-statistic (95% CI)***		**R^2 (95% CI)***	
	**Model**	**Model + NfL**	**Model**	**Model + NfL**
	0.76 (0.59-0.94)	0.82 (0.68-0.96)†	0.18 (0-0.52)	0.28 (0.04-0.63)†

Analyses were performed on the non-imputed datasets of subcohorts based on the availability of serum samples at a given time point. Serum neurofilament light chain levels were transformed into age-standardised Z-scores.
GBS: Guillain-Barré syndrome, CI: confidence interval, (s)NfL: (serum) neurofilament light chain, MRC: Medical Research Council.
* optimism-corrected, † $p<0.05$, [a] linear odds ratio.

Supplementary Table 5: Model performance statistics for the (modified) Erasmus Guillain-Barré syndrome Outcome Score with serum neurofilament light chain added before and after removal of clinical predictors.

	C-statistic* (95% CI)	R^2 (95% CI)
**EGOS + sNfL at week 1 (prediction for week 26)**		
Age + Preceding diarrhoea + GBS disability score at week 2 + sNfL at week 1[a]	0.88 (0.82-0.94)	0.44 (0.29-0.62)
Age + GBS disability score at week 2 + sNfL at week 1[a]	0.88 (0.82-0.94)[†]	0.45 (0.30-0.62)[†]
Preceding diarrhoea + GBS disability score at week 2 + sNfL at week 1[a]	0.88 (0.82-0.94)[†]	0.45 (0.29-0.63)[†]
Age + Preceding diarrhoea + sNfL at week 1[a]	0.75 (0.63-0.88)	0.28 (0.09-0.50)
GBS disability score at week 2 + sNfL at week 1[a]	0.88 (0.81-0.94)[†]	0.46 (0.30-0.62)[†]
Age + sNfL at week 1[a]	0.76 (0.63-0.88)	0.29 (0.11-0.50)
Preceding diarrhoea + sNfL at week 1[a]	0.73 (0.61-0.87)	0.28 (0.09-0.50)
**mEGOS at week 1 + sNfL at entry (prediction for week 4)**		
Age + Preceding diarrhoea + MRC sum score at week 1 + sNfL at entry	0.87 (0.82-0.92)	0.51 (0.38-0.66)
Age + MRC sum score at week 1 + sNfL at entry	0.87 (0.82-0.92)[†]	0.52 (0.39-0.66)[†]
Preceding diarrhoea + MRC sum score at week 1 + sNfL at entry	0.86 (0.81-0.92)[†]	0.50 (0.37-0.64)[†]
Age + Preceding diarrhoea + sNfL at entry	0.68 (0.60-0.76)	0.13 (0.03-0.27)
MRC sum score at week 1 + sNfL at entry	0.87 (0.81-0.92)[†]	0.50 (0.38-0.64)[†]
Age + sNfL at entry	0.68 (0.60-0.76)	0.13 (0.03-0.28)
Preceding diarrhoea + sNfL at entry	0.64 (0.56-0.73)	0.08 (0.00-0.21)

Analyses were performed in the imputed dataset of patients with available serum neurofilament light chain levels at the corresponding time point. CI: confidence interval, (m)EGOS: modified Erasmus Guillain-Barré syndrome Outcome Score, (s)NfL: (serum) neurofilament light chain, GBS: Guillain-Barré syndrome, MRC: Medical Research Council.
* optimism-corrected, [a] non-linear, [†] performs equally to the full model with neurofilament light chain added.
