# Supplementary material for: Neurofilament light chain improves clinical prognostic models for Guillain-Barré syndrome
Source: J Neurol Neurosurg Psychiatry. 2025 May 2;96(11):e336046. doi: 10.1136/jnnp-2025-336046 (PMC12573411; doi:10.1136/jnnp-2025-336046)
Supplement: online supplemental file 1 [file jnnp-96-11-s002.pdf]

PROTOCOL

Second **I**VIg **D**ose in **G**uillain-**B**arré **S**yndrome patients with poor prognosis (**SID-GBS trial**)

Erasmus MC
Prof. dr. P.A. van Doorn, neurologist
Dr. B.C. Jacobs, neurologist-immunologist
Prof. dr. E.W. Steyerberg, epidemiologist
Drs. C. Walgaard, resident in neurology

Johns Hopkins Hospital, Baltimore USA
Prof. dr. D. R. Cornblath, neurologist

Dutch GBS Study group

Subsidizing parties:
Sanquin, Amsterdam, the Netherlands
Prinses Beatrix Fonds

Prof. dr. P.A. van Doorn
Erasmus MC, dept. of Neurology
's-Gravendijkwal 230
3015 CE Rotterdam
+31.10.7033780
p.a.vandoorn@erasmusmc.nl

PROTOCOL TITLE
'**S**econd **IVIg** **D**ose in **G**uillain-**B**arré **S**yndrome patients with poor prognosis'

**Protocol ID**	SID2009
**Short title**	SID-GBS trial
**Dutch title**	Tweede immunoglobulinen (IVIg) kuur voor Guillain-Barré patiënten met een slechte prognose.
**Version**	Version 8
**ABR number**	26512
**Date**	September 9th 2009
**Sponsor / coordinating investigator**	Prof. dr. P.A. van Doorn<br>Erasmus MC, dept. of Neurology<br>'s-Gravendijkwal 230<br>3015 CE Rotterdam<br>+31.10.7033780<br>p.a.vandoorn@erasmusmc.nl
**Co-investigator**	Drs. C. Walgaard<br>Erasmus MC, dept. of Neurology<br>Dr. Molewaterplein 50-60<br>3015 GE Rotterdam<br>+31.10.7044209<br>c.walgaard@erasmusmc.nl
**Principal investigator site 1:**	Prof. dr. P.A. van Doorn, neurologist, Erasmus MC, Rotterdam
**Sub investigator site 1:**	Mw. C. Walgaard, MD neurology, Erasmus MC, Rotterdam
**Sub investigator site 1:**	Dr. B.C. Jacobs, neurologist, Erasmus MC, Rotterdam
**Principal investigator site 2:**	Dr. R. ten Houten, neurologist, Medisch Centrum Alkmaar, Alkmaar
**Principal investigator site 3:**	Mw. I.M. Bronner, neurologist, Flevoziekenhuis, Almere
**Principal investigator site 4:**	Dr. A. Hovestadt, neurologist, Meander MC, Amersfoort
**Principal investigator site 5:**	Mw. Dr. D.S.M. Molenaar, neurologist, Amstelland ziekenhuis, Amstelveen
**Principal investigator site 6:**	Mw. Dr. A.J. van der Kooi, neurologist, AMC, Amsterdam
**Principal investigator site 7:**	Prof. Dr. J.J. Heimans, neurologist, VU medisch

	centrum, Amsterdam
**Principal investigator site 8:**	I.N. van Schaik, neurologist, OLVG, Amsterdam
**Principal investigator site 9:**	Dr. G.J. Jöbsis, neurologist, Slotervaart ziekenhuis, Amsterdam
**Principal investigator site 10:**	Dr. W.H.J.P. Linssen, neurologist, Sint Lucas Andreas ziekenhuis, Amsterdam
**Principal investigator site 11:**	Mw. D. Skallebaek, neurologist, Gelre ziekenhuis, Apeldoorn
**Principal investigator site 12:**	Mw. D.G. Oenema, neurologist, Wilhelmina ziekenhuis, Assen
**Principal investigator site 13:**	P.J.I.M. Berntsen, neurologist, Lievensberg ziekenhuis, Bergen op Zoom
**Principal investigator site 14:**	W.D.M. van der Meulen, neurologist, Rode Kruis Ziekenhuis, Beverwijk
**Principal investigator site 15:**	Dr. M. Stevens, neurologist, Tergooi ziekenhuizen, Blaricum
**Principal investigator site 16:**	Dr. D. Pröpper, neurologist, Maasziekenhuis Pantein, Boxmeer
**Principal investigator site 17:**	W.M.J.H. Grosveld, neurologist, Reinier de Graaf ziekenhuis, Delft
**Principal investigator site 18:**	Dr. M.J.P. Garssen, neurologist, Jeroen Bosch ziekenhuis, Den Bosch
**Principal investigator site 19:**	Dr. K. Jellema, neurologist, MC Haaglanden, Den Haag
**Principal investigator site 20:**	Dr. P.W. Wirtz, neurologist, HagaZiekenhuis, Den Haag
**Principal investigator site 21:**	P.C.L.A. Lambregts, neurologist, Bronovo ziekenhuis, Den Haag
**Principal investigator site 22:**	D.Q. Ngo, neurologist, Deventer ziekenhuis, Deventer
**Principal investigator site 23:**	Dr. U.A. Badrising, neurologist, Stichting van Weel-Bethesda ziekenhuis, Dirksland
**Principal investigator site 24:**	Dr. R.P. Kleijweg, neurologist, Albert Schweitzer ziekenhuis, Dordrecht

**Principal investigator site 25:**	Dr. M.C. de Rijk, neurologist, Catharina ziekenhuis, Eindhoven
**Principal investigator site 26:**	Dr. P.J.A.M. Brouwers, neurologist, Medisch Spectrum Twente, Enschede
**Principal investigator site 27:**	Th.J.M. Breuer, neurologist, St Anna ziekenhuis, Geldrop
**Principal investigator site 28:**	Mw. Dr. A. Ruitenberg, neurologist, Oosterscheldeziekenhuis, Goes
**Principal investigator site 29:**	R.B. Alting van Geusau, neurologist, Beatrix ziekenhuis, Gorinchem
**Principal investigator site 30:**	Mw. J. Schuuring, neurologist, Groene Hart ziekenhuis, Gouda
**Principal investigator site 31:**	J.C.H. van Oostrom, neurologist, UMCG, Groningen
**Principal investigator site 32:**	Dr. R.J.O. van der Ploeg, neurologist, Martini ziekenhuis, Groningen
**Sub-investigator site 32:**	Dr. S.M. Schade van Westrum, neurologist, Martini ziekenhuis, Groningen
**Principal investigator site 33:**	Mw. H.M.E. Bienfait, neurologist, Kennemer Gasthuis, Haarlem
**Principal investigator site 34:**	Mw. H.J.G. Dieks, neurologist, Rupke-Zweers ziekenhuis, Hardenberg
**Principal investigator site 35:**	D.J. Hofstee, neurologist, St. Jansdal ziekenhuis, Harderwijk
**Principal investigator site 36:**	Mw. E.L. van der Kooi, neurologist, Medisch Centrum Leeuwarden
**Sub-investigator site 36:**	Mw. J. Krudde, neurologist, Medisch Centrum Leeuwarden
**Principal investigator site 37:**	Mw. Dr. R. van Koningsveld, neurologist, Elkerliek Ziekenhuis, Helmond
**Principal investigator site 38:**	P.G. Oomes, neurologist, Bethesda ziekenhuis, Hoogeveen
**Principal investigator site 39:**	T.C. van der Ree, neurologist, Westfries Gasthuis, Hoorn

**Principal investigator site 40:**	Dr. J.J.G.M Verschuuren, neurologist, LUMC, Leiden
**Principal investigator site 41:**	Mw. Dr. M.A. Hoving, neurologist, Diaconessenhuis Leiden
**Principal investigator site 42:**	R.J. Groen, neurologist, MCH Antoniushove, Leidschendam
**Principal investigator site 43:**	R.J.J. Tans, neurologist, IJsselmeerziekenhuizen, Lelystad
**Principal investigator site 44:**	Mw. Dr. C.G. Faber, neurologist, MUMC, Maastricht
**Principal investigator site 45:**	E.J.W. Keuter, neurologist, Diaconessenhuis Meppel
**Principal investigator site 46:**	Mw. Dr. M.F.G. van der Meulen, neurologist, St. Antonius ziekenhuis, Nieuwegein
**Principal investigator site 47:**	Prof. dr. B.G.M. van Engelen, neurologist, UMC St. Radboud, Nijmegen
**Sub-investigator site 47:**	Dr. N. van Alfen, neurologist, UMC St. Radboud, Nijmegen
**Principal investigator site 48:**	Dr. G.W. van Dijk, neurologist, Canisius Wilhelmina ziekenhuis, Nijmegen
**Principal investigator site 49:**	Dr. C.P. Zwetsloot, neurologist, Waterland ziekenhuis, Purmerend
**Principal investigator site 50:**	Mw. A.A.J.G.M. Schyns-Soeterboek, neurologist, Laurentius ziekenhuis, Roermond
**Principal investigator site 51:**	L. van Hooff, neurologist, Franciscus ziekenhuis, Roosendaal
**Principal investigator site 52:**	Mw. F.H. Vermeij, neurologist, Sint Franciscus Gasthuis, Rotterdam
**Principal investigator site 53:**	J.P.A. Samijn, neurologist, Maasstadziekenhuis, Rotterdam
**Principal investigator site 54:**	H.J. Vroon, neurologist, Havenziekenhuis, Rotterdam
**Principal investigator site 55:**	C.J. Gijsbers, neurologist, Vlietland ziekenhuis, Schiedam
**Principal investigator site 56:**	Dr. J.W.M. ter Berg, neurologist, Orbis Medisch Centrum, Sittard
**Principal investigator site 57:**	P.J.H.W. Jansen, neurologist, Antonius ziekenhuis,

	Sneek
**Principal investigator site 58:**	R.D. Oedit, neurologist, Ruwaard van Putten ziekenhuis, Spijkenisse
**Principal investigator site 59:**	Dr. A.T. Portman, neurologist, Refaja ziekenhuis, Stadskanaal
**Principal investigator site 60:**	Mw. Dr. T.A. Hoogendoorn, neurologist, Ziekenhuis Rivierenland, Tiel
**Principal investigator site 61:**	Dr. L.H. Visser, neurologist, Sint Elisabeth ziekenhuis, Tilburg
**Principal investigator site 62:**	Mw. M. Wohlgemuth, neurologist, Twee Steden Ziekenhuis, Tilburg
**Principal investigator site 63:**	Dr. W.L. van der Pol, neurologist, UMCU, Utrecht
**Principal investigator site 64:**	Mw. W. Westrate, neurologist, Diakonessenhuis, Utrecht
**Principal investigator site 65:**	Mw. Dr. F.H.H. Linn, neurologist, Centraal Militair Hospitaal, Utrecht
**Principal investigator site 66:**	J. Lion, neurologist, Bernhoven ziekenhuis, Veghel
**Principal investigator site 67:**	R.H.J. Medaer, neurologist, St Jans Gasthuis, Weert
**Principal investigator site 68:**	J.S. Straver, neurologist, Zuwe Hofpoort ziekenhuis, Woerden
**Principal investigator site 69:**	A. Koppenaal, neurologist, Zaans Medisch Centrum, Zaandam
**Principal investigator site 70:**	S.J. Mellema, neurologist, Isala klinieken, Zwolle
**Sub-investigator site 70:**	Dr. G. de Jong, neurologist, Isala klinieken, Zwolle.
**Subsidizing parties**	Sanquin Plasma Products<br>Medical Department<br>Plesmanlaan 125<br>1066 CX Amsterdam<br>P.F.W. Strengers, MD, medical advisor<br>Tel: +31-20 -5123239<br>I. Kleine Budde, PhD, trial coordinator<br>i.kleinebudde@sanquin.nl<br>Tel: +31-20 -5123239<br>Fax: +31-20-5123914<br><br>Prinses Beatrix Fonds

**Independent physician(s)**	Prof. Dr. R.Q. Hintzen, neurologist-immunologist<br>‘s-Gravendijkwal 230<br>3015 CE Rotterdam<br>+31.10.7033780
**Laboratory sites**	Sanquin Diagnostics<br><br>Laboratory of the corresponding hospital
**Coordinator immunological and serological studies**	Dr. B.C. Jacobs, neurologist-immunologist<br>Erasmus MC<br>Dr. Molewaterplein 50-60<br>3015 GE Rotterdam
**Pharmacy**	Pharmacy of the corresponding hospital<br><br>Mw. Sonja Kwadijk-De Gijssel, pharmacist, Clinical Trials

PROTOCOL SIGNATURE SHEET:

‘Second **I**VIg **D**ose in **G**uillain-**B**arré **S**yndrome patients with poor prognosis’

**Name**	**Signature**	**Date**
**Principal investigator / project leader / sponsor:**<br>Prof. dr. P.A. van Doorn, neurologist<br>Erasmus MC, Rotterdam		
**Head of the department Neurology:**<br>Prof. dr. P.A.E. Sillevis Smitt, neurologist<br>Erasmus MC, Rotterdam		

TABLE OF CONTENTS

LIST OF ABBREVIATIONS AND RELEVANT DEFINITIONS 11
SUMMARY 12
1. INTRODUCTION AND RATIONALE..13
2. OBJECTIVES 17
3. STUDY DESIGN 18
4. STUDY POPULATION 21
4.1. Population (base) 21
4.2. Inclusion criteria 21
4.3. Exclusion criteria 21
4.4. Sample size calculation 22
5. TREATMENT OF SUBJECTS...23
5.1 Investigational product / treatment 23
5.2 Use of co-intervention..23
6. INVESTIGATIONAL MEDICINAL PRODUCT 24
6.1. Name and description of investigational medicinal product 24
6.2. Summary of findings from non-clinical studies 24
6.3. Summary of findings from clinical studies 24
6.4. Summary of known and potential risks and benefits 24
6.5. Description and justification of route of administration and dosage 24
6.6. Dosages, dosage modifications and method of administration 25
6.7. Preparation and labelling of Investigational Medicinal Product 25
6.8. Drug accountability..25
7. METHODS..26
7.1. Study parameters/endpoints 26
7.1.1. Main study endpoint...26
7.1.2. Secondary study parameters/endpoints 26
7.1.3. Other study parameters...26
7.2. Randomization, blinding and treatment allocation.......................................27
7.3. Study procedures 27
7.4. Withdrawal of individual subjects...28
7.5. Replacement of individual subjects after withdrawal...................................28
7.6. Follow-up of subjects withdrawn from treatment 28
7.7. Premature termination of the study...28
8. SAFETY REPORTING 30
8.1. Section 10 WMO event...30
8.2. Adverse and serious adverse events (SAE)..30
8.3. Suspected unexpected serious adverse reactions (SUSAR).........................30
8.4. Annual safety report...31
8.5. Follow-up of adverse events...31
8.6. Data Safety Monitoring Board (DSMB) 31
9. STATISTICAL ANALYSIS..32
10. ETHICAL CONSIDERATIONS 33
10.1. Regulation statement..33
10.2. Recruitment and consent 33
10.3. Objection by minors or incapacitated subjects...33
10.4. Benefits and risks assessment, group relatedness......................................34
10.5. Compensation for injury..34
11. ADMINISTRATIVE ASPECTS AND PUBLICATION 35
11.1. Handling and storage of data and documents...35

11.2. Amendments....35
11.3. Annual progress report....36
11.4. End of study report....36
11.5. Public disclosure and publication policy....36
12. REFERENCES....37
APPENDIX A....39

LIST OF ABBREVIATIONS AND RELEVANT DEFINITIONS

**ABR**	**ABR form (General Assessment and Registration form) is the application form that is required for submission to the accredited Ethics Committee (ABR = Algemene Beoordeling en Registratie)**
**AE**	**Adverse Event**
**AR**	**Adverse Reaction**
**CA**	**Competent Authority**
**CCMO**	**Central Committee on Research Involving Human Subjects**
**CTCM**	**Clinical Trial Centre Maastricht**
**DSMB**	**Data Safety Monitoring Board**
**EU**	**European Union**
**EudraCT**	**European drug regulatory affairs Clinical Trials GCP Good Clinical Practice**
**IB**	**Investigator's Brochure**
**IENF**	**Intraepidermal nerve fiber**
**METC**	**Medical Research Ethics Committee (MREC); in Dutch: Medisch Ethische Toetsing Commissie (METC)**
**PI**	**Principal Investigator**
**(S)AE**	**(Serious) Adverse Event**
**SPC**	**Summary of Product Characteristics (in Dutch: officiële productinfomatie IB1-tekst)**
**Sponsor**	**The sponsor is the party that commissions the organisation or performance of the research, for example a pharmaceutical company, academic hospital, scientific organisation or investigator. A party that provides funding for a study but does not commission it is not regarded as the sponsor, but referred to as a subsidising party.**
**SUSAR**	**Suspected Unexpected Serious Adverse Reaction**
**WBP**	**Personal Data Protection Act (in Dutch: Wet Bescherming Persoonsgevens)**
**WMO**	**Medical Research Involving Human Subjects Act (in Dutch: Wet Medisch-wetenschappelijk Onderzoek met Mensen)**

SUMMARY

Rationale: Guillain-Barré syndrome (GBS) is the most frequent cause of acute neuromuscular weakness in the Western world. GBS patients have a variable prognosis, 20-30% needs mechanical ventilation, 20% is unable to walk after 6 months and 3% dies. Using a simple scoring system it is possible to accurately predict which patient has a poor prognosis. GBS patients with a poor prognosis may benefit from a second course of IVIg.

Objective: To determine whether a second IVIg course in GBS patients with a poor prognosis improves functional outcome after 4 weeks. Secondary outcomes include functional outcome after 8, 12 and 26 weeks, mechanical ventilation, length of hospital and ICU admission, occurrence of TRF's, mortality and blood IgG levels.

Study design: A double-blind randomized placebo-controlled trial design will be used in selected patients with a poor prognosis. In patients with a good prognosis the study will have an observational design.

Study population: GBS patients of 6 years and older, who have an indication for IVIg treatment.

Intervention: Patients with a poor prognosis according to the prediction model (mEGOS) will be randomized to receive a second IVIg course or placebo.

Main study parameters/endpoints: The main study endpoint is functional outcome on the GBS disability scale 4 weeks after start of the first IVIg course. Other endpoints include functional outcome after 26 weeks.

Nature and extent of the burden and risks associated with participation, benefit and group relatedness: The participant will be physically examined at 7 standardized time-points, mostly in combination with the standard patient care. Extra blood and CSF (extra spinal tab is not requested) will be used for trial purposes. An EMG will be performed in the standard clinical work-up. A throat swab will be requested. Serious adverse effects of IVIg are rare (details in SPC). It is anticipated that also in children benefits will outweigh the risks of participating in this treatment trial. Standard treatment in children is the same as in adults, long-term prognosis is better as in adults; however 25% is still not symptom free at a median of 228 days in a large observational study.

1. INTRODUCTION AND RATIONALE

Guillain-Barré syndrome (GBS) is an immune mediated peripheral polyradiculoneuropathy characterized by rapid onset flaccid paresis and sensory disturbances with a very heterogenic distribution in clinical characteristics and in prognosis. Some patients develop mild limb paresis, whereas others develop oculomotor, facial, bulbar, respiratory muscle and limb paralysis and remain bed bound for several months. GBS is the most frequent cause of acute neuromuscular weakness in the Western world[1]. It affects 1.2 persons per 100,000 per year[2]. Intravenous immunoglobulin (IVIg) and plasma exchange (PE) are shown to be effective in patients with GBS[3-6]. These studies primarily assessed the proportion of patients that improved 4 weeks after onset of this treatment. Nowadays, IVIg (2 g/kg in 2-5 days) has become standard treatment for patients with GBS who are unable to walk unaided and still within the first 2 weeks from onset of weakness[4, 7, 8]. The outcome of GBS after 6 or 12 months however has not, or only marginally been improved[1, 6]. Approximately 20% are still disabled after 6 months, mechanical ventilation is needed in 20-30% and 3%-10% of the patients die. Patients with severe GBS and poor prognosis may need additional or more aggressive therapy to recover. Careful selection of patients eligible for extra therapy is important, because extra treatment entail added complication risk and costs. Multiple clinical, electrophysiology, serological and laboratory factors have been identified as predictors for poor outcome. These include older age[9-11], rapid disease progression[11], disability grade[10, 12, 13], preceding diarrhea/Campylobacter jejuni serology[9, 10, 12], absence of antecedent upper respiratory tract infection[12, 13], axonal EMG[12, 13] and anti-GM1 antibodies[14, 15]. A recent study showed that the prognosis of individual GBS patients can accurately be predicted based on three simple clinical factors that can easily be obtained early during the course of disease[10]. The selection of patients for the RCT phase of this SID-GBS study is based on this prognostic model.

GBS can affect persons at virtually every age, standard treatment for children is the same as for adults (2 g IVIg/kg administered in 2-5 days). A large prospective, multicenter study in children showed that only 75% was symptom free at last follow-up (median of 228 days)[16]. This underlines the importance of better treatment strategies, also in this subgroup of patients. Children of 6 years and older were also included in the two randomized controlled treatment trials on which the EGOS model was based[10]. Since it is likely that children potentially may also benefit from a second IVIg dose, we will include children from 6 years and older in this therapeutic study.

Rationale for the study

There are several arguments suggesting that a second IVIg course can be effective in patients with a poor prognosis;

1. It is known that about 10% of GBS patients only have short-lasting improvement after a single IVIg course (‘treatment-related clinical fluctuation’), a second dose of IVIg is then followed by functional improvement, suggesting that one IVIg course in these patients is insufficient [17]
2. A second course of IVIg is suggested to be effective in a small uncontrolled series of severe unresponsive GBS patients[18]

3. Additionally, recent data from our group show that patients with a relatively minor increase in serum IgG level after IVIg treatment recover significantly slower and fewer patients were able to walk unaided after 6 months (Kuitwaard, Ann Neurol in press).

Based on these findings, it is likely that at least a proportion of GBS patients may benefit from repeated courses of IVIg [6], especially the subgroup of patients with poor prognosis.

There are several arguments, however, for not treating all patients with a second IVIg course;

1. A proportion of GBS patients recover relatively well with the current treatment regime.
2. Thrombo-embolic (TE) complications are rare but are regularly described with the use of IVIg, most likely because of a rise in plasma viscosity [19-25]. A recent case-control study found that the risk of TE complications was elevated in patients with multiple cardiovascular risk factors[26]. Neurologic patients may be more prone to this complication because of their impaired mobility [20].
3. Presently the consumption of IVIg is increasing at an annual rate of 5% in Europe and 11% in the USA. A relative shortage of IVIg has been witnessed in recent years. In addition, IVIg is used in an off-label manner for nearly 100 conditions. High costs and limited resources will require targeted use of IVIg[21, 27].

We think it is justified to treat GBS patients with high risk of poor outcome with a second IVIg course because in those patients the benefits outweigh the risks.

Erasmus GBS Outcome Scale (EGOS)

Recently, a prognostic model (EGOS) was developed that accurately predicts long-term outcome in GBS. The model was developed using a large number of GBS cases obtained from the Erasmus MC GBS databank, and the accuracy was confirmed in an independent large cohort of GBS patients[10]. One of the advances of the EGOS is that it needs only 3 simple and easy obtainable clinical features in the acute stage of GBS. Advanced age, presence of diarrhea ≤ 4 weeks before onset of GBS and high GBS disability score 2 weeks after start of first treatment were identified to be related with poor functional outcome after 6 months[10]. By using this simple and well-evaluated prognostic EGOS model, it is possible to select GBS patients with a poor prognosis that may benefit from a second IVIg dosage. Poor prognosis is defined as not being able to walk unaided (GBS disability score of 3 or higher).

Advanced modified EGOS (mEGOS)

In the SID-GBS study we will use a modified version of the EGOS (table and figure 1).

Table 1: The modified Erasmus GBS Outcome Score (mEGOS) chart.

	Categories	Score
Age at onset (years)	≤ 40	0
	41-60	1
	> 60	2
Diarrhoea (≤ 4 weeks before onset of weakness)	Absence	0
	Presence	1
MRC sumscore (1 week after entry)	51 – 60	0
	41 – 50	3
	31 – 40	6
	0 – 30	9
modified Erasmus GBS Outcome Score (mEGOS)		0 – 12

In the modified version we will obtain data from the GBS patient 1 week after start of first IVIg treatment, because it is important to start a second course within the first 2 weeks of the illness when nerve damage might still be reversible. To construct the mEGOS we used the MRC sumscore (a score often used in GBS research) (table 4) at 1 week instead of the GBS disability score. The MRC sumscore consists of muscle force of 6 muscle pairs in both sides of the body, the MRC sumscore ranges from 0 (paralysis) to 60 (maximal muscle force). At one week (after onset of IVIg treatment) the MRC sumscore has a better predictive power than the GBS disability score. A second modification is that we will use the model to predict outcome at 4 weeks. A four week endpoint is usually the primary endpoint in clinical treatment trials in GBS. The GBS disability score is the scale generally used as outcome measure in GBS trials. Therefore we will use the GBS disability scale at four weeks as primary outcome in this trial.

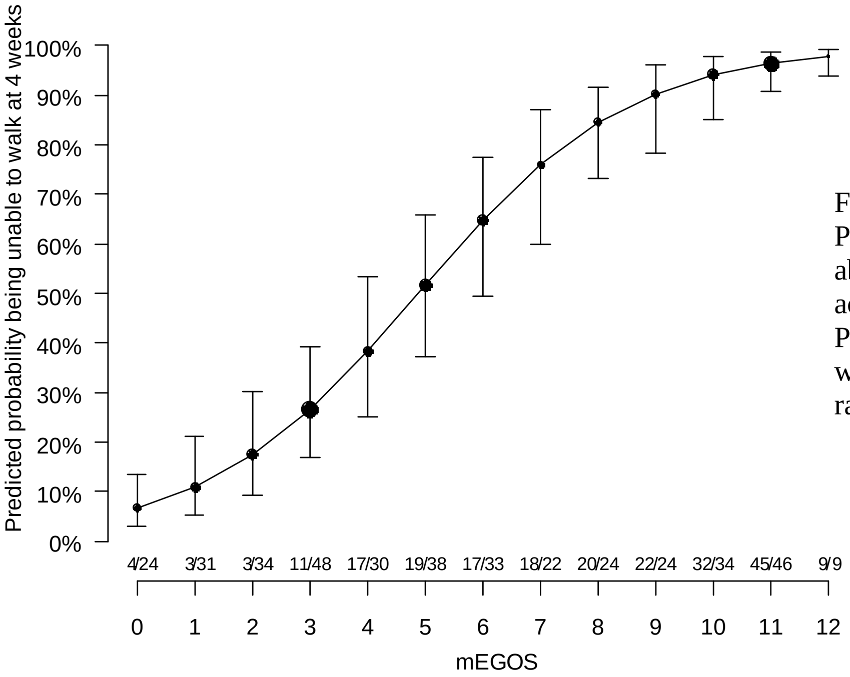

Figure 1
Predicted probability being able to walk at 4 weeks according to mEGOS. Patients with mEGOS 6-12 will be selected for randomization.

This mEGOS has a very good predictive power (Area Under the Receiver Operating Characteristic (ROC) Curve (AUC) = 0.87) for prediction of outcome after 4 weeks, indicating very good discriminative ability. In the patient group with a mEGOS of ≥ 6 (48% of total) 85% had a poor prognosis (being unable to walk unaided) after 4 weeks and 35% after 6 months (figure 2).

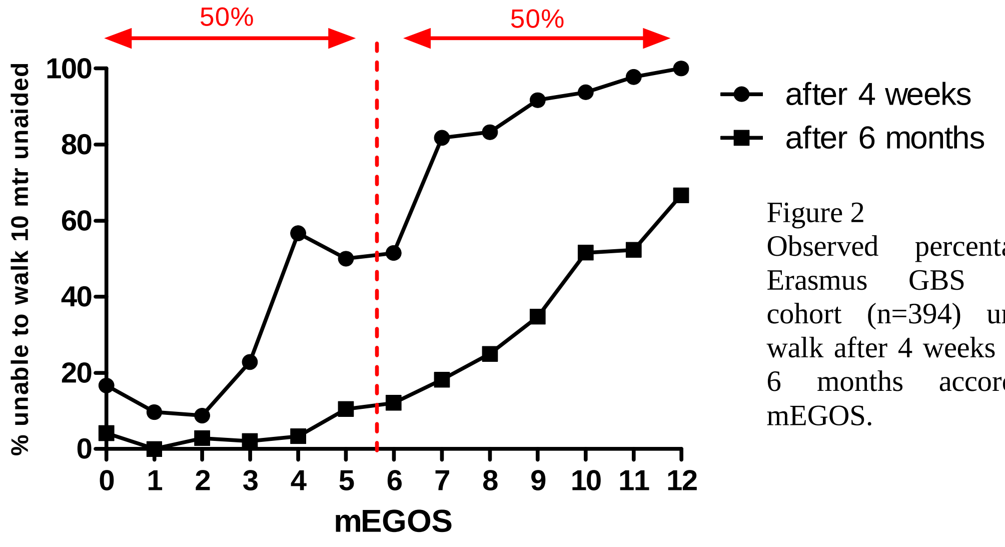

Figure 2
Observed percentages in Erasmus GBS databank cohort (n=394) unable to walk after 4 weeks and after 6 months according to mEGOS.

Also mEGOS accurately predicts long-term outcome after 6 months of follow-up, with a good calibration (figure 3) and discrimination (AUC = 0.84).

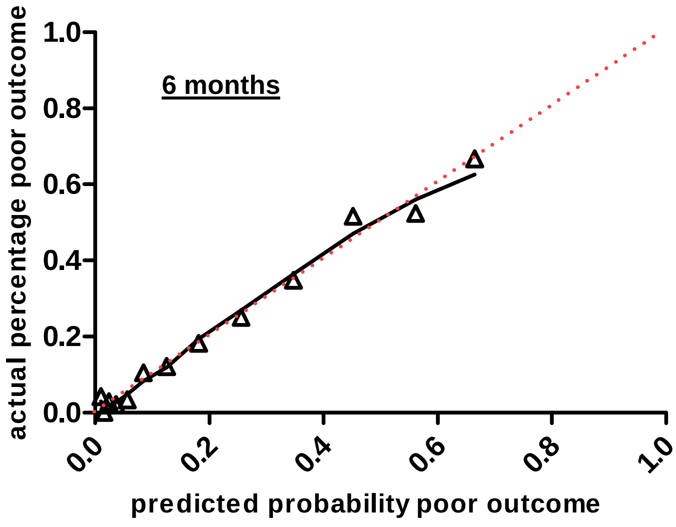

Figure 3
Calibration of the mEGOS model in the development data (n=394) for prediction of poor outcome after 6 months. The dotted line from 0.0 to 1.0 indicates perfect calibration. Triangles indicate the probabilities in grouped patients with similar predicted risks. A non-parametric, smoothed curve indicates the relation between predicted probability and observed frequency of poor outcome after 6 months.

Using mEGOS of six as a cutoff point we will select approximately 50% of patients. Those patients with a poor prognosis will be randomized to get a second IVIg course or placebo.

Hypothesis
GBS patients with the poorest prognosis (mEGOS 6-12) may benefit from a second course of IVIg when administered within the first weeks after onset of disease, when nerve damage is most likely still reversible.

2. OBJECTIVES

Primary objective
To determine whether a second IVIg dosage in GBS patients with a poor prognosis improve functional outcome after 4 weeks.

Secondary objectives

To investigate whether:

- a second IVIg dosage in GBS patients with a poor prognosis improve functional outcome or muscle strength after 8, 12 and 26 weeks.

- a second IVIg dosage in GBS patients with a poor prognosis lowers the percentage of patients needing artificial ventilation, lower the time (number of days) on respirator or time on the intensive care.

- a second IVIg dosage in GBS patients with a poor prognosis reduces the time to hospital discharge.

- a second IVIg dosage in GBS patients with a poor prognosis reduce the chance of secondary deterioration due to treatment-related fluctuations (TRF[†]).

- patients treated with a second IVIg dosage develop more complications possibly related to the second IVIg treatment.

- a second IVIg dosage in GBS patients with a poor prognosis lowers the percentage of patients that die because of GBS.

- the serum IgG increase after the first IVIg dosage is lower in patients with a poor prognosis.

- serum IgG increases further (and to what extent) after administration of a second IVIg dosage.

† A TRF is defined as (1) improvement in the GBS disability score of at least one grade or improvement in the MRC sum score (0-60) of more than 5 points after completion of therapy, followed by a worsening in the GBS disability score of at least one grade or a decrease in the MRC sumscore of more than 5 points within the first months after onset of disease or (2) stabilization of the clinical course for more than 1 week after completion of therapy, followed by a worsening of at least one grade of the GBS disability score or more than 5 points on the MRC sumscore.

3. STUDY DESIGN

A double-blind randomized placebo-controlled trial design will be used in selected patients with a poor prognosis. In patients with a good prognosis the study will have an observational design.

- All GBS patients in need of IVIg treatment, according to the treating neurologist, in a standard dosage of 2 g/kg in 2-5 consecutive days are potentially eligible for this study after obtaining informed consent.
- When patients sign 'Informed consent' they principally agree to be randomized to get a second IVIg dose or placebo when having a poor prognosis at day 7 and to be followed up for 6 months.
- Patients with the poorest prognosis based upon the modified EGOS (mEGOS 6-12) after the first IVIg course will be randomized to get a second course of IVIg (Nanogam®) in a dosage of 8 ml/kg (=0,4 g/kg) for 5 days or placebo (GPO®) in a dosage of 8 ml/kg for 5 days in a blinded fashion.
- mEGOS must preferentially be assessed 7 days after start of first IVIg course, with a range to 8 or 9 days. Trial medication needs then to be started within 24 hours when indicated according to the mEGOS score (see figure 4).
- Patient follow-up will be 6 months.
- Total patient inclusion will end when 44 patients with a poor prognosis received a second IVIg dosage.
- After covariate adjustment, to deal with variation between patients in baseline risk and to increase statistical power, data of the placebo group and the intervention group will be compared.

This is shown in the flowchart (table 2).

Figure 4

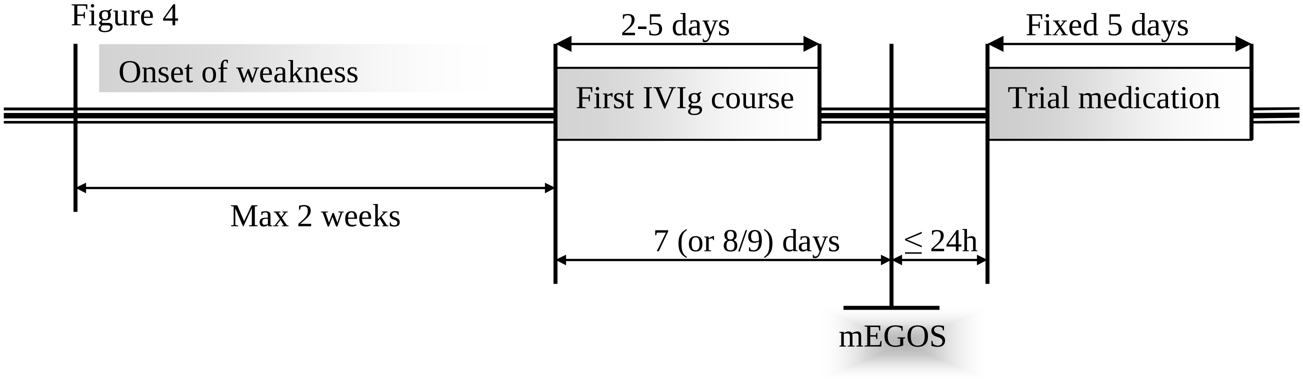

Inclusion procedure

We contacted neurologists of 93 hospitals in the Netherlands by information letter. We also will contact neurologists formally being member of the Dutch GBS study group, additionally this study will be brought under the attention during national meetings of neurologists. When neurologists are willing to cooperate in this study local feasibility in their hospital will be checked according to GCP guidelines and neurologists will be

provided with pocket size inclusion cards. These cards describe in – and exclusion criteria, checklists for the trial procedures and additional contact information.
When a GBS patient in need of IVIg treatment is admitted in one of the cooperating hospitals and in –and exclusion criteria are met the patient (or his / her legal representative) is asked to participate in this study.

Data collection

1. Questionnaires encompassing patient demographics, preceding events, indicators of disease severity, disease progression and outcome will be filled out at predefined standard time points by the PI and after monitoring will be send to the coordinating center at Erasmus MC.
2. Blood samples, CSF and a throat swap will be send to the coordinating center. The specimen will be analyzed at the Erasmus MC at the department of neuro-immunology and microbiology. In addition DNA will be stored for future gene polymorphism studies. We expect that the collected samples will be of interest for future research in the next decade, so we will store the material for 15 years, when informed consent is obtained.
3. An EMG is performed in the standard work-up of GBS patients. The PI will be asked to perform an EMG following predefined guidelines to enable classification of electrophysiological data according to Hadden[28] as indicative of primary demyelinating disease, equivocal, primary axonal, unexcitable and normal. Guidelines were developed in collaboration with the clinical neurophysiology department of the Erasmus MC.
4. After discharge from the hospital, patients will be asked to visit the outpatient clinic of the local hospital for follow-up. When a patient is fully recovered according to the GBS disability score, the MRC sum score and the ONLS score at 3 months, they will be discharged from further follow-up, presuming GBS is a monophasic disease.
5. Patients of 18 years or older included in hospitals in Rotterdam, will be asked to have a skin biopsy (3 mm punch) to determine intraepidermal nerve fibre (IENF) density. Yet, unpublished research from our group showed prognostic value of IENF density in skin biopsies. We aim to expand this study. Skin biopsy for determination of IENF density is a standard - minimal invasive - procedure requiring local anesthetics and resulting in a small scar. Estimated time 10 minutes. The skin biopsy protocol includes a skin biopsy at the distal leg and a lumbar paraspinal biopsy. The biopsies will be taken in the first week of admission and after 6 months. Skin biopsies are additional to the standard protocol, patients can decide to participate in the SID-GBS study without giving approval for taking skin biopsies. Therefore patient information and informed consent forms are strictly separate.

Further follow-up is requested from all patients in the informed consent letter to enable further studies involving long-term outcome. Improvement of neurological deficit is known to occur until many years after onset of GBS[29], especially in severely affected patients. Long-term follow-up (at 1 or 2 years) will be executed by semi-standardized telephone interviews.

Table 2; flowchart

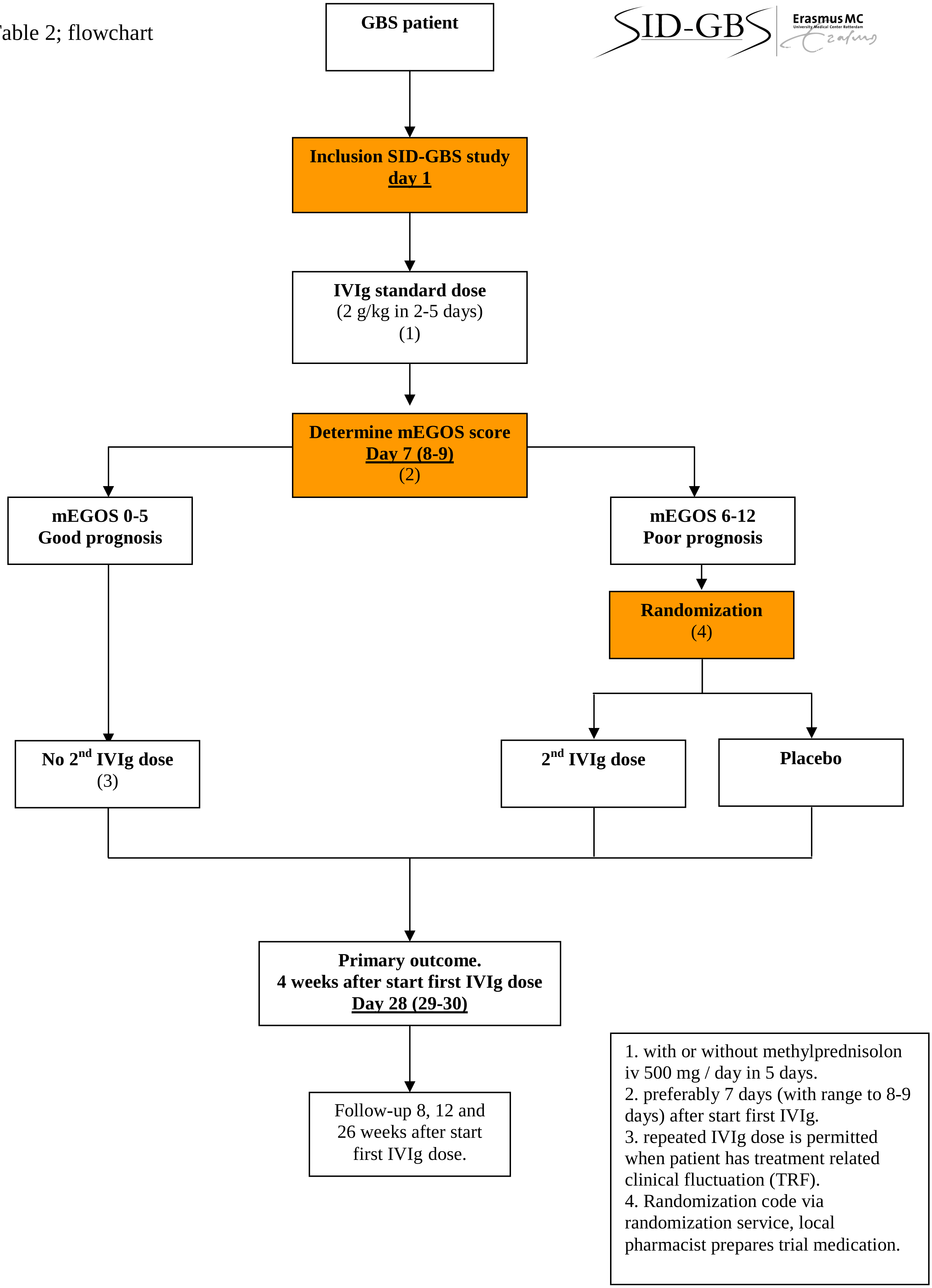

4. STUDY POPULATION

4.1. Population (base)

This study will be executed in the Netherlands. The annual incidence of GBS in the Netherlands is 1.2 per 100.000 persons [2], so there will be around 200 newly diagnosed GBS patients each year. At present 67 hospitals in the Netherlands are willing to participate in this study.

The inclusion period will start around October 2009 and is expected to end 3 years later. In this period we expect to include the needed 174 patients, of which 50% (87 patients) will be randomized to receive a second dosage of Nanogam® or placebo.

4.2. Inclusion criteria

A. To enter this GBS study

- Patients are diagnosed with GBS[30].
- There is an indication to start IVIg (irrespective of co-treatment with methylprednisolon (MP)) therapy:
  1. Patient is unable to walk unaided for >10 meter (grade 3, 4 or 5 of the GBS disability scale)
  <u>or</u>
  2. There is otherwise an indication to start IVIg (with or without MP) treatment according to the treating neurologist.
- Onset of weakness due to GBS is less than 2 weeks ago.
- Signed informed consent.

B. To be randomized in the second IVIg dose phase (RCT), patients must fulfill the following criteria:

- First IVIg (with or without MP) treatment with Nanogam® started within 2 weeks from onset of weakness.
- IVIg treatment has been 2g/kg administered in 2-5 days.
- Poor prognosis based upon the modified EGOS (mEGOS 6-12) at day 7 after start of first IVIg treatment.

4.3. Exclusion criteria

A. To enter this GBS study

- Age less than 6 years.
- Patient known to have a severe allergic reaction to properly matched blood products or plasma products.
- Pregnancy or breastfeeding.
- Patient known to have a selective IgA deficiency.
- Patient shows clear clinical evidence of a polyneuropathy caused by e.g. diabetes mellitus (except mild sensory), alcoholism, severe vitamin deficiency, porphyria.

- Patient received immunosuppressive treatment (e.g. azathioprine, cyclosporine, mycofenolaatmofetil, tacrolimus, sirolimus or > 20 mg prednisolon daily) during the last month.
- Patient known to have a severe concurrent disease, like malignancy, severe cardiovascular disease, AIDS, severe CARA.
- Inability to attend follow-up during 6 months.

B. Relative contra-indications for second IVIg dose:

- Patients known to have severe kidney dysfunction (GFR below 40 ml/min).
- Pre-existing risk factors of thrombo-embolic complications or severe ischemic heart disease.

These patient groups run a greater risk (although they are still rare) to develops serious complications like acute tubular necrosis and thrombo-embolic events. To prevent this they should be pre-treated with fluids and infusion rate of the trial medication must be adjusted.

4.4. Sample size calculation

- We will select patients with poorest prognosis (mEGOS 6-12), this corresponds with approximately 50% of the population.
- Calculations showed that the prognosis of this group (mEGOS 6-12) is poor both at 4 weeks (85% unable to walk unaided) and at 26 weeks (35% unable to walk unaided).
- We presume that a second dose of IVIg in patients with poor prognosis (mEGOS 6-12) will raise the percentage of patients improving on the GBS disability score at 4 weeks with 15-25%. For power analysis for our primary outcome we used an improvement of 20%.
- Assuming a 20% improvement in unadjusted data and with a dichotomous outcome (being able to walk unaided versus being unable to walk unaided) we will need (α=0.05 and power=0.80) 290 patients in total (145 poor and 145 good prognosis).
- The power increase through covariate adjustment and the use of the ordinal outcome may be expected to result in a reduction in required sample size of 40 to 50% [31]. This leads to a required sample size of 60% of 290 patients = 174 patients.
- Based on previous calculations 50% of 174 patients (n=87) will have a poor prognosis. This group of 87 patients will be randomized to receive a second course of IVIg (n=44) or placebo (n=44).
- We expect that a sample size of 174 patients is well feasible with approximately 3 years of accrual.

5. TREATMENT OF SUBJECTS

5.1 Investigational product / treatment

Intravenous immunoglobulin product
Details on Nanogam® 50 mg/ml (RVG 31627) can be found in the SPC and IB.

Placebo
Details on GPO® (“Gepasteuriseerde Plasma-eiwit Oplossing”, pasteurised plasma protein solution) (RVG16911) can be found in the SPC and IB.

5.2 Use of co-intervention

All medication used during the study will be recorded on the case report form.

6. INVESTIGATIONAL MEDICINAL PRODUCT

6.1. Name and description of investigational medicinal product

The medicinal product under investigation is immunoglobulins (IVIg) (Nanogam®). In this trial the medicinal product is used in a different regiment, which is in a repeated fashion in the same dosage as the first course (2g/kg in 5 days) for patients with severe GBS.
As placebo we will use GPO. This solution contains 40g/l protein, of which 95% is albumin. A summary of findings and information about those medicinal products can be found in the SPC and IB.

6.2. Summary of findings from non-clinical studies

Details on Nanogam and GPO can be found in the SPC.

6.3. Summary of findings from clinical studies

Details on Nanogam and GPO can be found in the SPC.

6.4. Summary of known and potential risks and benefits

Nanogam and GPO are produced by Sanquin Plasma products, Amsterdam. Nanogam and GPO are manufactured from plasma of voluntary, non-remunerated donors. The viral safety of the products have been established by donor selection and screening for HIV1-2, HTLV I/II and hepatitis B and C, by plasma pool screening for HIV, HCV, HBV and Parvo B19 at RNA /DNA level, and by effective inactivation and/or removal of viruses with several physico-chemical methods in the manufacturing process.
The most common adverse effects occur soon after infusion with Nanogam and can include chills, headache, fever, vomiting, allergic reactions, nausea, arthralgia, low blood pressure and moderate low back pain. Side effects are rare during or after infusion of albumin products. Mild reactions such as flush, urticaria, fever, and nausea are usually temporarily and often associated with infusion rate.

6.5. Description and justification of route of administration and dosage

The first (or only) IVIg course in a dosage of 2g/kg in 2-5 days is standard treatment for GBS patients who are unable to walk and still within the first two weeks from onset of weakness, independent of age of the patient or severity of disease. Dose finding studies with IVIg in GBS has never been performed. In some hospitals in the Netherlands neurologists already give a second course of IVIg in patients with severe disease, although the efficacy has never been proven. The second course of IVIg in this trial will be in the same fashion as the first course (0,4 g/kg BW on five consecutive days) because neurologists are familiar with this treatment regiment and it is also used in patients with TRF. Nanogam is manufactured in a 5% solution (50 gram/litre), hence it follows that 0.4 g/kg Nanogam is 8 ml Nanogam/kg BW. Accordingly, 8 ml/kg BW on five consecutive

days of the placebo, GPO, will be administered. GPO and Nanogam will be infused using identical infusion systems.

6.6. Dosages, dosage modifications and method of administration

Not applicable

6.7. Preparation and labelling of Investigational Medicinal Product

Every participating hospital owns a buffering stock of IVIg (Nanogam®) and GPO (shelf lives of 3 and 4 years). When a patient is eligible for entering the second RCT phase of this study, the patient will be randomized to get a second IVIg course or placebo. The local pharmacist will prepare the trial medication according to the randomization allocation.

6.8. Drug accountability

Not applicable

7. METHODS

7.1. Study parameters/endpoints

7.1.1. Main study endpoint

GBS disability score at 4 weeks after start of first IVIg course. The full range of scores will be considered as an ordinal outcome scale. In analyzing we will use a proportional odds model. (Extent of) improvement on this ordinal scale will be compared between groups.

7.1.2. Secondary study parameters/endpoints

- Percentage of patients that improve:
  at least 1, 2, 3 or 4 points on the GBS disability score at 4, 8, 12 and 26 weeks,
  at least 4, 8 or 12 points on MRC sum score (ranging from 0-60) at 4, 8, 12 and 26 weeks,
  at least 2, 4 or 6 points on ONLS score (ranging from 0-12) at 4, 8, 12 and 26 weeks.
- Percentage of patients needing artificial ventilation.
- Time (number of days) on respirator.
- Time (number of days) on intensive care unit.
- Percentage of patients that die because of GBS.
- Time (number of days) to hospital discharge.
- Percentage of patients with secondary deterioration due to treatment-related fluctuations (TRF).
- Development of complications possibly related to a second IVIg course.
- Serum IgG levels at 5 different time points.

7.1.3. Other study parameters

On admission a detailed anamnesis should be taken relating to blood transmittable viral infections, other relevant diseases (relevant surgical interventions in the past five years, history of blood transmittable viral infections, anaphylactic reactions to the use of plasma products) and the use of other medication.

- To correct for known prognostic factors (age, preceding diarrhea, positive serology of different micro-organisms, antibodies against gangliosides) we will ask questions about antecedent events, as diarrhea and upper respiratory tract infection and perform laboratory measurements (serology against *Campylobacter jejuni*, cytomegalovirus (CMV), Epstein-Barr virus (EBV), *Mycoplasma pneumonia* and IgG, IgM and IgA antibodies against GM1, GD1a and GQ1b.) IENF density in skin biopsies (additional).

7.2. Randomization, blinding and treatment allocation

All GBS patients in need of IVIg treatment are potentially eligible for this study. All participants will be evaluated after first IVIg treatment en scored following the modified EGOS model. Patients with a poor prognosis according to this model will be randomized to receive a second IVIg dosage or placebo. For that a randomization website is used (see the signed agreement with CTCM). A computer-generated list of random assignments (block randomization per site) is prepared in advance. When a PI registers a patient to the website a box number is generated and an email with this box number will be send automatically to the local pharmacist (and the central pharmacy of the Erasmus MC, the coordinating investigator, the co-investigator, Sanquin and the registering PI). The box number corresponds to the randomization list and the local pharmacist will prepare the study medication according to the randomization allocation. The PI will prescribe the trial medication in the same dosage as the first treatment (2 g/kg administered in 5 consecutive days). The coordinating investigator, the co-investigator, the PI and the trial coordinator of Sanquin as well as the patient will be blinded to the treatment.
Blinding will be broken in case of a Serious Adverse Event considering the severity of the adverse event and the likeliness of a relation to IVIg treatment after conference between the investigators, Sanquin and the treating neurologist. The treating neurologist will then be unblinded and is responsible for treatment of the possible side effects of the IVIg treatment.
Blinding will not be broken in case of a TRF, a TRF is defined as (1) improvement in the GBS disability score of at least one grade or improvement in the MRC sum score (0-60) of more than 5 points after completion of the first IVIg course, followed by a worsening in the GBS disability score of at least one grade or a decrease in the MRC sumscore of more than 5 points within the first months after onset of disease or (2) stabilization of the clinical course for more than 1 week after completion of the first IVIg course, followed by a worsening of at least one grade of the GBS disability score or more than 5 points on the MRC sumscore. When a TRF occurs during the trial medication course, the trial medication will be stopped and an open IVIg course will be started, as this is standard treatment for a TRF. This decision should always be taken after consultation with the trial coordination.

7.3. Study procedures

When patients are included in the study they will undergo the following extra procedures;

- Throat swaps
- Blood collection
  Blood collection will take place before start of standard IVIg treatment (visit 1), after standard IVIg treatment (visit 2), after two weeks (visit 3), after 4 weeks (visit 4) and after 3 months (visit 6). Mostly it will be possible to collect blood for the study simultaneously with vena punctures performed in the scope of the medical work-up.
- CSF collection
  At admission virtually all patients undergo a lumbar puncture as part of the standard medical workup; extra CSF will be collected for the SID-GBS study. In this way there is no need for an extra spinal puncture. For the study a small

sample (5 cc) of CSF is sufficient. If no lumbar puncture was performed for various reasons, no lumbar puncture will be performed in the scope of this study.

- EMG examination generally is comparable with the situation outside a study, but may be more extensive in some patients. This depends on the local procedures in the participating hospitals. An EMG guideline is developed in a way that a minimum set of nerves is tested to enable classification of electrophysiological data according to Hadden[28].
- <u>Additional for patients of 18 years and older and included in a hospital in Rotterdam.</u>
  Two times two skin biopsies for determination of IENF density. Punch biopsy of 3 mm 10 cm above the lateral malleolus and paraspinal after local anesthesia with 1% lidocaine. Skin biopsy for determination of IENF density is a minimal invasive procedure. Estimated time 10 minutes. There is a very small risk of getting an infection. Some people get a scar at the site of the biopsy (often less than 3mm, conform the size of the punch biopsy).

7.4. Withdrawal of individual subjects

Subjects can be withdrawn from the study at any time for any reason if they wish to do so without any consequences. The PI can decide to withdraw a subject from the study for urgent medical reasons. In all cases, the reasons why patients are withdrawn must be recorded on the CRF and in the patients' medical record.

7.5. Replacement of individual subjects after withdrawal

Not applicable

7.6. Follow-up of subjects withdrawn from treatment

For all subjects who are prematurely withdrawn from treatment, the reason will be documented carefully. The patients who had at least one dose of study medication will at least be included in the safety evaluation.

7.7. Premature termination of the study

Severe complications attributable to IVIg are rare. Yet, clinical evidence of an association between thrombo-embolic complications (myocardial infarction, stroke, lung embolism, deep venous thrombosis) and IVIg treatment exists. This association is probably attributable to the relative increase in blood viscosity because of the high influx of immunoglobulins. It is imaginable that a second dosage of IVIg in a short period further increases the chance of developing such a severe complication, especially in patients with pre-existing risk factors of thrombo-embolic complications (older age, hypertension, diabetes mellitus, history of vascular disease or thrombo-embolic illnesses, patients with acquired or inborn pre-thrombotic disease, patients with long term immobilization, deep hypovolemic state and patients who have illnesses which cause increased viscosity of the blood). Extra precautions will be taken in patients with pre-existing risk factors for thrombo-embolic complications and patients with kidney function disturbances. When

significantly more severe complications occur most likely attributable to the second IVIg dosage the study will be prematurely terminated.

8. SAFETY REPORTING

8.1. Section 10 WMO event

In accordance to section 10, subsection 1, of the WMO, the investigator will inform the subjects and the reviewing accredited METC if anything occurs, on the basis of which it appears that the disadvantages of participation may be significantly greater than was foreseen in the research proposal. The study will be suspended pending further review by the accredited METC except insofar as suspension would jeopardize the subjects' health. The investigator will take care that all subjects are kept informed.

8.2. Adverse and serious adverse events (SAE)

Adverse events are defined as any undesirable experience occurring to a subject during a clinical trial, whether or not considered related to the investigational drug. All adverse events reported spontaneously by the subject or observed by the investigator or his staff will be recorded.

A serious adverse event is any untoward medical occurrence or effect that at any dose;

- results in death
- is life threatening (at the time of the event)
- requires hospitalization or prolongation of existing inpatients' hospitalization*
- results in persistent or significant disability or incapacity
- is a congenital anomaly or birth defect
- is a new event of the trial likely to affect the safety of the subjects, such as an unexpected outcome of an adverse reaction, lack of efficacy of an IMP used for the treatment of a life threatening disease, major safety finding from a newly completed animal study, etc

**all patients in this trial are hospitalized, this is not considered to be a serious adverse event.*

All SAE's will be reported to the accredited METC that approved the protocol, according to the requirement of that METC.

8.3. Suspected unexpected serious adverse reactions (SUSAR)

Adverse reactions are all untoward and unintended responses to an investigational product related to any dose administered.

Unexpected adverse reactions are adverse reaction, of which the nature, or severity, is not consistent with the applicable product information (Investigator's Brochure).

The sponsor will report expedited the following SUSARs to the METC:

- SUSARs that have arisen in the clinical trial that was assessed by the METC

- SUSARs that have arisen in other clinical trial of the same sponsor and with the same medicinal product, and that could have consequences for the safety of the subjects involved in the clinical trial that was assessed by the METC.

The remaining SUSARs are recorded in an overview list (line-listing) that will be submitted once every half year to the METC. This line-listing provides an overview of all SUSARs from the study medicine, accompanied by a brief report highlighting the main points of concern.

The sponsor will report expedited all SUSARs to the competent authority, the Medicine Evaluation Board and the competent authorities in other member states.

The expedited reporting will occur not later than 15 days after the sponsor has first knowledge of the adverse reactions. For fatal or life threatening cases the term will be maximal 7 days for a preliminary report with another 8 days for completion of the report.

8.4. Annual safety report

In addition to the expedited reporting of SUSARs, the sponsor will submit, once a year throughout the clinical trial, a safety report to the accredited METC, competent authority, Medicine Evaluation Board and competent authorities of the concerned Member States.
This safety report consists of:

- A list of all suspected (unexpected and expected) serious adverse reactions, along with an aggregated summary table of all reported serious adverse reactions, ordered by organ system
- A report concerning the safety of the subjects, consisting of a complete safety analysis and an evaluation of the balance between the efficacy and the harmfulness of the medicine under investigation.

8.5. Follow-up of adverse events

All adverse events will be followed until they have abated, or until a stable situation has been reached. Depending on the event, follow op may require additional tests or medical procedures as indicated, and/or referral to the general physician or a medical specialist.

8.6. Data Safety Monitoring Board (DSMB)

Dr. Diederik W.J. Dippel, neurologist / epidemiologist and Prof. Dr. Rogier Q. Hintzen, neurologist / neuro-immunologist at Erasmus MC, will serve as members of the independent data- and safety monitoring committee. The committee assesses the progress of the trial, the safety data and the efficacy endpoints and records whether to continue, modify or stop the trial every 6 months and will report their findings to the steering-committee (prof. dr. Pieter A. van Doorn, dr. Bart C. Jacobs, prof. dr. Ewout W. Steyerberg and prof. dr. David R. Cornblath)

9. STATISTICAL ANALYSIS

The analysis will be by intention-to-treat. A simple first analysis is to compare outcomes from the 44 patients who received a second IVIg course with the 44 patients who received placebo.

Covariate adjustment

We will use covariate adjustment, which is an established approach to deal with variation between patients in baseline risk and to increase statistical power in clinical phase III trials. (Roozenbeek et al. Critical Care Medicine, in press). Using covariate adjustment we can also compare outcomes from the 44 patients who receive a second IVIg course with the other included patients (n=130) with good and poor prognosis.
Unadjusted analysis can be expressed by the following formula, in which α indicates the intercept and β represents the regression coefficient for the treatment:

$$\text{Log odds (favorable)} = \alpha + \beta * \text{treatment}$$

The covariate-adjusted model uses modified EGOS after first IVIg treatment as well as the treatment variable:

$$\text{Log odds (favorable)} = \alpha + \beta * \text{treatment} + \beta 1 * \text{mEGOS}$$

The increase in statistical power of covariate adjustment depends on the predictive strength of the baseline characteristics; this is difficult to quantify a priori, but the modified EGOS has a very good predictive power (AUC=0.874).

Proportional Odds model

Furthermore, we can consider the full GBS disability score at 4 weeks as an ordinal outcome, rather than dichotomize as ≤2 vs >2. Analysis will then be with a proportional odds regression model. The proportional odds model provides a more sensitive analysis than would be possible by arbitrarily dichotomizing the outcome variable and does so without imposing unverifiable assumptions regarding the structure of the data[32]. The disadvantage is that we have to make the assumption of proportional odds, i.e. that the treatment effect (as an odds ratio) is identical across all possible cut-offs for the GBS disability score. This assumption will be assessed by a test for heterogeneity of effect across cut-offs.

Further exploratory analyses will be performed using appropriate statistical summary measures (medians, means, standard deviations, standard errors, interquartile ranges) and statistical tests (both parametric and non-parametric).

10. ETHICAL CONSIDERATIONS

10.1. Regulation statement

The study will be conducted according to the principles of the Declaration of Helsinki (59th WMA General Assembly, Seoul, October 2008) and according to the latest revised version of the Medical Research Involving Human Subjects (WMO). The procedures set out in this study protocol are designed to ensure that the sponsor and investigator abide by the principles of the GCP guidelines of the European Community (ICH topic E6, CPMP/ICH/135/95, Directive 2001/20/EC) and the Declaration of Helsinki (in the latest revised version) in the conduct, evaluation and documentation of this study. Copies of the ICH-GCP-Guidelines and the Declaration of Helsinki are included in the Investigator's Study File.
The protocol has to be approved by the Medical Ethical Committee of the local Hospital before a patient can enter the study.

10.2. Recruitment and consent

All GBS patients admitted to the participating hospitals will be asked to participate in this study by their treating neurologist or PI. He or she will inform the patients about the study using the patient information letter. We developed a special patient information letter for children of 12-17 years of age. Contact information of the researchers and the independent physician is given in the patient information letter and intended participants are encouraged to contact them. All patients will be given opportunity to ask questions and allowed sufficient time to consider the information provided. The patient's signed and dated informed consent will be obtained and filed before conducting any procedure specifically for the study. The investigators will store the original, signed informed consent form. A copy of the signed informed consent form will be given to the patient.

10.3. Objection by minors or incapacitated subjects

Objection by minors or incapacitated will be dealt with in conformity with the code of conduct 'Gedragscode bij verzet van minderjarigen die deelnemen aan medisch-wetenschappelijk onderzoek' (in English; code of conduct in case of objection by minors taking part in medical research). This code of conduct was accepted on May 21, 2001 by a conference of the Board of the Dutch Society of Paediatrics (Nederlandse Vereniging voor Kindergeneeskunde, NVK) and is published on the website of the CCMO (Dutch Competent Authority). When a trial participant objects against any act he or she is submitted to according the trial protocol the study should not take place in that particular participant (WMO, art. 4, lid 2). In conference with the parents or caretakers an assessment is made about suspected reactions in the child. Possible objection of the minor and which behaviour is defined as objection will be discussed with the parents or caretakers of the minor. Code of conduct in case of objection of the individual minor will be indicated.

10.4. Benefits and risks assessment, group relatedness

Benefit of IVIg in GBS patients, who were unable to walk unaided and who were started on IVIg within the first 2 weeks after onset of weakness, is clearly demonstrated and IVIg is at present the standard treatment in GBS[6, 33]. Very limited information is available concerning the optimal dosage of IVIg. For reasons described earlier we expect benefit of a second dosage IVIg in patients with poor prognosis; however this has never been proven. IVIg treatment is usually well tolerated; minor side effects can include headache, chest pain, myalgia, nausea, or fatigue presumably mediated by the release of pro-inflammatory cytokines in susceptible patients. Many of these symptoms are easily managed by slowing down the infusion rate or the use of co-medication with non-steroidal antiphlogistics. Severe complications are rare and include arterial or venous thrombosis (such as pulmonary embolism, stroke, and myocardial infarction), anaphylaxis, most likely in the case of allergic or hereditary IgA deficiency (which is a contra-indication for IVIg therapy and consequently for this study); and renal disturbances, that rarely has been described in patients already known with insufficient renal function. A second IVIg course theoretically can have side-effects due to the load of protein and increased blood viscosity. Finally IVIg is expensive and there appears to be a potential world-wide shortage. More intensive treatment applied in a targeted population has advantage both from a medical and socio-economical point of view.

10.5. Compensation for injury

The investigator has a liability insurance which is in accordance with article 7, subsection 6 of the WMO. The sponsor (also) has an insurance which is in accordance with the legal requirements in the Netherlands (Article 7 WMO and the Measure regarding Compulsory Insurance for Clinical Research in Humans of 23th June 2003). This insurance provides cover for damage to research subjects through injury of death caused by the study.

1. €450.000,-- (i.e. four hundred and fifty thousand Euro) for death or injury for each subject who participates in the research;
2. €3500.000,-- (i.e. three million five hundred thousand Euro) for death or injury for all subjects who participate in the research;
3. €5.000.000,-- (i.e. five million Euro) for the total damage incurred by the organisation for all damage disclosed by scientific research for the sponsor as ‘verrichter’ in the meaning of said Act in each year of insurance coverage.

The insurance applies to the damage that becomes apparent during the study or within 4 years after the end of the study.

11. ADMINISTRATIVE ASPECTS AND PUBLICATION

11.1. Handling and storage of data and documents

When a GBS patient is included in the study he or she will be assigned a unique number. Name, address and date of birth will be stored separately from the study data. Data will be recorded on CRFs and entered in the database twice to prevent data entry faults. The database will contain rules to prevent the entry of invalid data. Data will be entered regularly to prevent missing data being overlooked. Independent monitoring will be established to ensure that the study is conducted, recorded and reported in accordance with the protocol and GCP. The investigators, members of the Health Inspection and members of the Medical Ethical Committee have access to personal data. Research data will be handled with due observance of the Dutch Law for Protection of Personal data (Wet Bescherming Persoonsgegevens) and the privacy statement of the Erasmus MC. With informed consent research data will be stored during 15 years. Human material will be stored non-traceable for 15 years, when informed consent is obtained. We expect that the stored material will be very valuable for future research.

Study Monitoring

Sanquin Plasma Products has developed monitoring and auditing procedures. Monitors or delegates of Sanquin Plasma Products will monitor the site, in order to comply with GCP guidelines. For this study, the expected average monitoring frequency is 6 months, or more frequent, if necessary, by personal visit. The pharmacy will be visited once a year or more frequent, if necessary. The hospital laboratory will be visited when required. Checking of the CRFs for completeness and clarity, and cross-checking with source documents in the presence of the investigator - giving due consideration to data protection and medical confidentiality - will be required, and the investigator assures Sanquin Plasma Products of support at all times. This will be necessary to monitor the progress of the study. The regulatory authorities and/or the sponsor’s Clinical Quality Assurance Group may also wish to carry out such source data checks and/or inspections/audits.

11.2. Amendments

A ‘substantial amendment’ is defined as an amendment to the terms of the METC application, or to the protocol or any other supporting documentation, that is likely to affect to a significant degree:

- The safety or physical or mental integrity of the subjects of the trial;
- The scientific value of the trial;
- The conduct or management of the trial; or
- The quality or safety of any intervention used in the trial.

All substantial amendments will be notified to the METC and to the competent authority.
Non-substantial amendments will not be notified to the accredited METC and the competent authority, but will be recorded and filed by the sponsor.

11.3. Annual progress report

The sponsor / principal investigator will submit a summary of the progress of the trial to the accredited METC once a year. Information will be provided on the date of inclusion of the first subject, numbers of subjects included and numbers of subjects that have completed the trial, serious adverse events/serious adverse reactions, other problems and amendments.

11.4. End of study report

The sponsor / principal investigator will notify the accredited METC and the competent authority of the end of the study within a period of 90 days. The end of the study is defined as the last patient’s last visit. Inclusion of patients will stop after 44 patients eligible for a second dosage IVIg have been included.
In case the study is ended prematurely, the sponsor will notify the accredited METC and the competent authority within 15 days, including the reasons for the premature termination.
Within one year after the end of the study, the investigator/sponsor will submit a final study report with the results of the study, including any publications/abstracts of the study, to the accredited METC and the Competent Authority.

11.5. Public disclosure and publication policy

This national trial will be conducted on behalf of the Dutch GBS study group. The results of this study will be published in the name of the SID-GBS study group. There will be a writing group, consisting out of the members of the Steering committee and researchers especially working on this study. Neurologists who include patients are members of the Dutch GBS study group, they will be listed as co-authors according to the numbers of patients included in their center.
Papers will be published conforming to CCMO guidelines. Any scientific communications drafted by the investigators should be sent to the Medical Department of Sanquin Plasma Products at least 30 days prior to submission to a scientific journal/meeting. Sanquin Plasma Products do not have the right to withhold the data from publication but can require changes with respect to issues regarding proprietary protection. If necessary, the authorities will be notified of the investigator’s name, address, qualifications and extent of involvement. By signing the study protocol, the investigators agree with the use for publication and information for medical and pharmaceutical professionals.

12. REFERENCES

1. Hughes, R.A. and D.R. Cornblath, *Guillain-Barre syndrome.* Lancet, 2005. 366(9497): p. 1653-66.
2. Van Koningsveld, R., et al., *Mild forms of Guillain-Barre syndrome in an epidemiologic survey in The Netherlands.* Neurology, 2000. 54(3): p. 620-5.
3. *Plasmapheresis and acute Guillain-Barre syndrome. The Guillain-Barre syndrome Study Group.* Neurology, 1985. 35(8): p. 1096-104.
4. van der Meche, F.G. and P.I. Schmitz, *A randomized trial comparing intravenous immune globulin and plasma exchange in Guillain-Barre syndrome. Dutch Guillain-Barre Study Group.* N Engl J Med, 1992. 326(17): p. 1123-9.
5. *Efficiency of plasma exchange in Guillain-Barre syndrome: role of replacement fluids. French Cooperative Group on Plasma Exchange in Guillain-Barre syndrome.* Ann Neurol, 1987. 22(6): p. 753-61.
6. Hughes, R.A., et al., *Immunotherapy for Guillain-Barre syndrome: a systematic review.* Brain, 2007. 130(Pt 9): p. 2245-57.
7. *Randomised trial of plasma exchange, intravenous immunoglobulin, and combined treatments in Guillain-Barre syndrome. Plasma Exchange/Sandoglobulin Guillain-Barre Syndrome Trial Group.* Lancet, 1997. 349(9047): p. 225-30.
8. van Koningsveld, R., et al., *Effect of methylprednisolone when added to standard treatment with intravenous immunoglobulin for Guillain-Barre syndrome: randomised trial.* Lancet, 2004. 363(9404): p. 192-6.
9. Hadden, R.D., et al., *Preceding infections, immune factors, and outcome in Guillain-Barre syndrome.* Neurology, 2001. 56(6): p. 758-65.
10. van Koningsveld, R., et al., *A clinical prognostic scoring system for Guillain-Barre syndrome.* Lancet Neurol, 2007. 6(7): p. 589-94.
11. Winer, J.B., R.A. Hughes, and C. Osmond, *A prospective study of acute idiopathic neuropathy. I. Clinical features and their prognostic value.* J Neurol Neurosurg Psychiatry, 1988. 51(5): p. 605-12.
12. *The prognosis and main prognostic indicators of Guillain-Barre syndrome. A multicentre prospective study of 297 patients. The Italian Guillain-Barre Study Group.* Brain, 1996. 119 (Pt 6): p. 2053-61.
13. Chio, A., et al., *Guillain-Barre syndrome: a prospective, population-based incidence and outcome survey.* Neurology, 2003. 60(7): p. 1146-50.
14. Annunziata, P., et al., *Association of anti-GM1 antibodies but not of anti-cytomegalovirus, Campylobacter jejuni and Helicobacter pylori IgG, with a poor outcome in Guillain-Barre syndrome.* J Neurol Sci, 2003. 213(1-2): p. 55-60.
15. Koga, M., et al., *Anti-GM1 antibody IgG subclass: a clinical recovery predictor in Guillain-Barre syndrome.* Neurology, 2003. 60(9): p. 1514-8.
16. Korinthenberg, R., J. Schessl, and J. Kirschner, *Clinical presentation and course of childhood Guillain-Barre syndrome: a prospective multicentre study.* Neuropediatrics, 2007. 38(1): p. 10-7.
17. Ruts, L., R. van Koningsveld, and P.A. van Doorn, *Distinguishing acute-onset CIDP from Guillain-Barre syndrome with treatment related fluctuations.* Neurology, 2005. 65(1): p. 138-40.

18. Farcas, P., et al., *Efficacy of repeated intravenous immunoglobulin in severe unresponsive Guillain-Barre syndrome.* Lancet, 1997. 350(9093): p. 1747.
19. Caress, J.B., et al., *The clinical features of 16 cases of stroke associated with administration of IVIg.* Neurology, 2003. 60(11): p. 1822-4.
20. Dalakas, M.C. and W.M. Clark, *Strokes, thromboembolic events, and IVIg: rare incidents blemish an excellent safety record.* Neurology, 2003. 60(11): p. 1736-7.
21. Gold, R., M. Stangel, and M.C. Dalakas, *Drug Insight: the use of intravenous immunoglobulin in neurology--therapeutic considerations and practical issues.* Nat Clin Pract Neurol, 2007. 3(1): p. 36-44.
22. Hefer, D. and M. Jaloudi, *Thromboembolic events as an emerging adverse effect during high-dose intravenous immunoglobulin therapy in elderly patients: a case report and discussion of the relevant literature.* Ann Hematol, 2005. 84(6): p. 411-5.
23. Katz, U. and Y. Shoenfeld, *Review: intravenous immunoglobulin therapy and thromboembolic complications.* Lupus, 2005. 14(10): p. 802-8.
24. Okuda, D., et al., *Arterial thrombosis induced by IVIg and its treatment with tPA.* Neurology, 2003. 60(11): p. 1825-6.
25. Vucic, S., et al., *Thromboembolic complications of intravenous immunoglobulin treatment.* Eur Neurol, 2004. 52(3): p. 141-4.
26. Caress, J.B., et al., *Case-control study of thromboembolic events associated with IV immunoglobulin.* J Neurol, 2009.
27. Bayry, J., M.D. Kazatchkine, and S.V. Kaveri, *Shortage of human intravenous immunoglobulin--reasons and possible solutions.* Nat Clin Pract Neurol, 2007. 3(3): p. 120-1.
28. Hadden, R.D., et al., *Electrophysiological classification of Guillain-Barre syndrome: clinical associations and outcome. Plasma Exchange/Sandoglobulin Guillain-Barre Syndrome Trial Group.* Ann Neurol, 1998. 44(5): p. 780-8.
29. Dhar, R., L. Stitt, and A.F. Hahn, *The morbidity and outcome of patients with Guillain-Barre syndrome admitted to the intensive care unit.* J Neurol Sci, 2008. 264(1-2): p. 121-8.
30. Asbury, A.K. and D.R. Cornblath, *Assessment of current diagnostic criteria for Guillain-Barre syndrome.* Ann Neurol, 1990. 27 Suppl: p. S21-4.
31. Murray, G.D.B.I., Lu J et al. , *Approaches to outcome analysis in traumatic brain injury trials.* J Neurotrauma, 2006. 23(5): p. 740-801.
32. Scott, S.C., M.S. Goldberg, and N.E. Mayo, *Statistical assessment of ordinal outcomes in comparative studies.* J Clin Epidemiol, 1997. 50(1): p. 45-55.
33. Elovaara, I., et al., *EFNS guidelines for the use of intravenous immunoglobulin in treatment of neurological diseases: EFNS task force on the use of intravenous immunoglobulin in treatment of neurological diseases.* Eur J Neurol, 2008. 15(9): p. 893-908.

APPENDIX A

Assessment scales

table 3: GBS disability score

GBS disability score

0	A healthy state
1	Minor symptoms and capable of running
2	Able to walk 10 metres or more without assistance but unable to run
3	Able to walk 10 metres across an open space with help (e.g. sticks, crutches, walker)
4	Bedridden or chairbound
5	Requiring assisted ventilation for at least part of the day
6	death

table 4: MRC sumscore

MRC sumscore

Sum of MRC scores from six bilaterally muscle groups, including shoulder abductors, elbow flexors, wrist extensors, hip flexors, knee extensors and foot dorsiflexors, ranging from 60 (normal strength) to 0 (quadriplegic). The MRC sum of an individual muscle ranges from 0-5:

0	No visible contraction
1	Visible contraction without movement of the limb
2	Active movement of the limb, but not against gravity
3	Active movement against gravity over (almost) the full range
4	Active movement against gravity and resistance
5	Normal strength

table 5: Overall Neuropathy Limitations Scale

<u>Overall Neuropathy Limitations Scale (ONLS)</u>

Instructions: The examiner should question and observe the patient in order to determine the answers to the following questions. Note should be made of any other disorder other than peripheral neuropathy which limits function at the foot of the page.

ARM SCALE

Does the patient have any symptoms in their hands or arms, eg tingling, numbness or weakness?

☐ yes ☐ no (if 'no', please go to legs section)

**Is the patient affected in their ability to:**	Not affected	Affected, but not prevented	Prevented
Wash and brush their hair	☐	☐	☐
Turn a key in a lock	☐	☐	☐
Use a knife and fork together (or spoon, if knife and fork not used)	☐	☐	☐
Do or undo buttons or zips	☐	☐	☐
Dress the upper part of their body (excluding buttons or zips)	☐	☐	☐

If all these functions are prevented can the patient make purposeful movements with their hands or arms? Yes ☐ No ☐ Not applicable ☐

ARM GRADE

0= Normal
1= Minor symptoms in one or both arms but not affecting any of the functions listed
2= Disability in one or both arms affecting but not preventing any of the functions listed
3= Disability in one or both arms preventing at least one but not all functions listed
4= Disability in both arms preventing all functions listed but purposeful movement still possible
5= Disability in both arms preventing all purposeful movements

SCORE = _____

LEG SCALE

	Yes	No	Not Applicable
Does the patient have difficulty running or climbing stairs?	☐	☐	☐
Does the patient have difficulty with walking? Does their gate look normal?	☐	☐	☐
How do they mobilise for about 10 metres (ie 33 feet)?			
- Without aid	☐	☐	☐
- With one stick or crutch or holding to someone's arm	☐	☐	☐
- With two sticks or crutches or one stick or crutch holding onto someone's arm or frame	☐	☐	☐
- With a wheelchair	☐	☐	☐
If they use a wheelchair, can they stand and walk 1 meter with the help of one person?	☐	☐	☐
If they cannot walk as above are they able to make some purposeful movements with their legs, eg reposition legs in bed?	☐	☐	☐
Does the patient use ankle foot orthoses/braces? (please circle)	☐	☐ If yes: right/left (please circle)	

LEG GRADE

0= Walking/climbing stairs/running not affected
1= Walking/climbing stairs/ running affected, but gait does not look abnormal
2= Walks independently but gait looks abnormal
3= Requires unilateral support to walk 10 metres (stick, crutch, one arm)
4= Requires bilateral support to walk 10 metres (sticks, crutches, crutch and arm or frame)
5= Requires wheelchair to travel 10 metres but able to stand and walk 1 metre with the help of one person
6= Restricted to wheelchair, unable to stand and walk 1 metre with the help of one person, but able to make some purposeful leg movements
7= Restricted to wheelchair or bed most of the day, unable to make any purposeful movements of the legs

SCORE = _____

Overall Neuropathy Limitation Scale = arm scale (range 0-5) + leg scale (range 0-7);
range 0 (no disability) and 12 (maximum disability)

TOTAL SCORE = _____

Is there any disorder, other than peripheral neuropathy, which affects the above functions?
Yes ☐ No ☐
If yes, please describe
